# P21-Activated Kinase Inhibitors FRAX486 and IPA3: Inhibition of Prostate Stromal Cell Growth and Effects on Smooth Muscle Contraction in the Human Prostate

**DOI:** 10.1371/journal.pone.0153312

**Published:** 2016-04-12

**Authors:** Yiming Wang, Christian Gratzke, Alexander Tamalunas, Nicolas Wiemer, Anna Ciotkowska, Beata Rutz, Raphaela Waidelich, Frank Strittmatter, Chunxiao Liu, Christian G. Stief, Martin Hennenberg

**Affiliations:** 1 Department of Urology, Ludwig-Maximilians University, Munich, Germany; 2 Department of Urology, Zhujiang Hospital, Southern Medical University, Guangzhou, China; Institute of Molecular and Cell Biology (IMCB), SINGAPORE

## Abstract

Prostate smooth muscle tone and hyperplastic growth are involved in the pathophysiology and treatment of male lower urinary tract symptoms (LUTS). Available drugs are characterized by limited efficacy. Patients’ adherence is particularly low to combination therapies of 5α-reductase inhibitors and α_1_-adrenoceptor antagonists, which are supposed to target contraction and growth simultaneously. Consequently, molecular etiology of benign prostatic hyperplasia (BPH) and new compounds interfering with smooth muscle contraction or growth in the prostate are of high interest. Here, we studied effects of p21-activated kinase (PAK) inhibitors (FRAX486, IPA3) in hyperplastic human prostate tissues, and in stromal cells (WPMY-1). In hyperplastic prostate tissues, PAK1, -2, -4, and -6 may be constitutively expressed in catecholaminergic neurons, while PAK1 was detected in smooth muscle and WPMY-1 cells. Neurogenic contractions of prostate strips by electric field stimulation were significantly inhibited by high concentrations of FRAX486 (30 μM) or IPA3 (300 μM), while noradrenaline- and phenylephrine-induced contractions were not affected. FRAX486 (30 μM) inhibited endothelin-1- and -2-induced contractions. In WPMY-1 cells, FRAX486 or IPA3 (24 h) induced concentration-dependent (1–10 μM) degeneration of actin filaments. This was paralleled by attenuation of proliferation rate, being observed from 1 to 10 μM FRAX486 or IPA3. Cytotoxicity of FRAX486 and IPA3 in WPMY-1 cells was time- and concentration-dependent. Stimulation of WPMY-1 cells with endothelin-1 or dihydrotestosterone, but not noradrenaline induced PAK phosphorylation, indicating PAK activation by endothelin-1. Thus, PAK inhibitors may inhibit neurogenic and endothelin-induced smooth muscle contractions in the hyperplastic human prostate, and growth of stromal cells. Targeting prostate smooth muscle contraction and stromal growth at once by a single compound is principally possible, at least under experimental conditions.

## Introduction

Patients with benign prostatic hyperplasia (BPH) are often characterized by voiding symptoms, caused by bladder outlet obstruction (BOO) due to abnormal prostate smooth muscle tone and prostate enlargement [1–3]. Besides other receptors, smooth muscle contraction in the prostate is induced by activation of α_1_-adrenoceptors, while hyperplastic growth is triggered by dihydrotestosteron [2, 3]. Consequently, α_1_-blockers or other medications are routinely applied for immediate improvement of symptom scores or urinary flow by relaxation of prostate smooth muscle, while prostate size may be reduced by 5α-reductase inhibitors to prevent progression, complications, and operation [3]. However, medical therapy is still hampered by insufficient efficacy and low patients’ adherence to medications [2, 4]. Combination therapies are required to reduce prostate smooth muscle tone and prostate size at once, but are associated with high discontinuation rates [2–4].

Limitations of α_1_-blockers may be explained by contributions of non-adrenergic mediators, which may induce prostate smooth muscle contraction even in the presence of α_1_-blockers [2]. Such non-adrenergic mediators, which increase prostate smooth muscle tone in parallel to α_1_-adrenoceptors, include endothelins and thromboxane A2 [2]. In addition, prostate enlargement may contribute to BOO besides contraction, and despite application of α_1_-blockers, as α_1_-blockers do not affect prostate size [3]. Thus, an ideal medication for treatment of voiding symptoms would address adrenergic and non-adrenergic smooth muscle contraction, plus prostate growth at once. Nowadays, a possible context between smooth muscle contraction and growth in the hyperplastic prostate has been assumed, but underlying molecular mechanisms are poorly understood [2, 5].

Recently, it became clear that previous models being used for more than one decade are insufficient to explain contraction of prostate smooth muscle [2]. Several studies uncovered the role of intracellular effectors, which are involved in control of prostate smooth muscle contraction besides Ca^2+^-, protein kinase C-, or Rho kinase-dependent pathways [2, 6–8]. Thereby, the monomeric GTPase Rac was identified as a critical mediator of smooth muscle contraction and stromal growth in the prostate [8]. Rac may act together with p21-activated kinases (PAKs) in different cell types [9, 10]. Although Rac signalling in the prostate turned out to be PAK-independent, this suggested for the first time a possible role of PAKs for smooth muscle contraction and growth in the prostate [8]. Nevertheless, effects of PAK inhibitors in the hyperplastic prostate have at present not been examined to the best of our knowledge.

PAKs are a group of serine/threonine kinases, being involved in a broad range of cellular functions, including regulation of cytoskeleton organization, smooth muscle contraction, neuronal function, or cell cycle [10–12]. PAKs may promote smooth muscle contraction in the airways, gastrointestinal tract, and cardiovascular system [13–21]. Besides this role for smooth muscle tone, PAKs may be involved in the control of cell cycle in airway and vascular smooth muscle cells [22–25]. In the prostate, PAK has been regarded in oncological context, but to the best of our knowledge not in the non-malignant organ [26–30]. However, based on its dual role for smooth muscle contraction and cell cycle of smooth muscle cells outside the lower urinary tract, it appears tempting to assume similar PAK functions in the prostate. Due to the contributions of smooth muscle contraction and growth in the prostate to voiding symptoms in millions of patients, understanding the molecular control of both processes and identification of their mediators is in fact attractive. Together, this prompted us to examine PAK expression and effects of PAK inhibitors in the hyperplastic human prostate, and in prostate stromal cells.

## Methods

### Human prostate tissues

Human prostate tissues were obtained from patients undergoing radical prostatectomy for prostate cancer, but without previous transurethral resection of the prostate (TURP). The research was carried out in accordance with the Declaration of Helsinki of the World Medical Association, and has been approved by the ethics committee of the Ludwig-Maximilians University, Munich, Germany (approval number 158–15). Informed written consent was obtained from the participants; data were analysed anonymously. Tissues were taken from the periurethral zone, while most prostate tumors are located to the peripheral zone [31, 32]. Tissue samples did not exhibit histological signs of neoplasia, cancer, or inflammation. BPH is present in ca. 80% of patients with prostate cancer [33, 34]. Samples were taken immediately after prostatectomy, following macroscopical examination by a pathologist. Organ bath studies were performed immediately after sampling, while samples for molecular analyses were shock frozen in liquid nitrogen and stored at -80°C.

### Real time polymerase chain reaction (PCR)

RNA from frozen prostate tissues was isolated using the RNeasy Mini kit (Qiagen, Hilden, Germany). For isolation, 30 mg of tissue was homogenized using the FastPrep^®^-24 system with matrix A (MP Biomedicals, Illkirch, France). RNA concentrations were measured spectrophotometrically. Reverse transcription to cDNA was performed with 1 μg of isolated RNA using the Reverse Transcription System (Promega, Madison, WI, USA). Real time- (RT-) PCR for PAK isoforms, and glyceraldehyde 3-phosphate dehydrogenase (GAPDH) was performed with a Roche Light Cycler (Roche, Basel, Switzerland) using primers provided by Qiagen (Hilden, Germany) as ready-to-use mixes, based on the RefSeq Accession numbers NM_001128620 for PAK1, NM_002577 for PAK2, NM_001128166 for PAK3, NM_001014831 for PAK4, NM_020341 for PAK5, NM_001128628 for PAK6, and NM_002046 for GAPDH. PCR reactions were performed in a volume of 25 μl containing 5 μl LightCycler^®^ FastStart DNA Master^Plus^ SYBR Green I (Roche, Basel, Switzerland), 1 μl template, 1 μl primer, and 18 μl water. Denaturation was performed for 10 min at 95°C, and amplification with 45 cycles of 15 sec at 95°C followed by 60 sec at 60°C. The specificity of primers and amplification was demonstrated by subsequent analysis of melting points, which revealed single peaks for each target. Results were expressed based on the number of cycles (Ct), at which the fluorescence signal exceeded a defined treshold.

### Western blot analysis

Frozen prostate tissues were homogenized in a buffer containing 25 mM Tris/HCl, 10 μM phenylmethanesulfonyl fluoride, 1 mM benzamidine, and 10 μg/ml leupeptine hemisulfate, using the FastPrep^®^-24 system with matrix A (MP Biomedicals, Illkirch, France). After centrifugation (20,000 g, 4 min), supernatants were assayed for protein concentration using the Dc-Assay kit (Biorad, Munich, Germany) and boiled for 10 min with sodium dodecyl sulfate (SDS) sample buffer (Roth, Karlsruhe, Germany). Samples of WPMY-1 cells were prepared as described below. Samples of prostate homogenates (20 μg/lane) or WPMY-1 cells (40 μg/lane) were subjected to SDS-polyacrylamide gel electrophoresis, and proteins were blotted on Protran^®^ nitrocellulose membranes (Schleicher & Schuell, Dassel, Germany). Membranes were blocked with phosphate-buffered saline (PBS) containing 5% milk powder (Roth, Karlsruhe, Germany) over night, and incubated with rabbit anti PAK1 (#2602) (New England Biolabs, Ipswich, MA, USA), mouse anti αPAK (sc-166887), goat anti γPAK (sc-7117), mouse anti γPAK (sc-373740), goat anti βPAK (sc-1871), mouse anti PAK3 (H00005063-M08) (Abnova, Taipei City, Taiwan), rabbit PAK4 (sc-28779), mouse anti PAK4 (sc-393367), rabbit PAK6 (sc-32857), mouse anti PAK6 (sc-393075), goat anti PAK5 (sc-22155), mouse anti PAK7 (MAB4696) (R&D Systems, Minneapolis, MN, (USA), mouse anti pan-cytokeratin (sc-8018), mouse anti calponin 1/2/3 (sc-136987), mouse anti prostate-specific antigen (PSA) (sc-7316), or mouse anti β-actin antibody (sc-47778) (if not other stated, all from Santa Cruz Biotechnology, Santa Cruz, CA, USA). Primary antibodies were diluted 1:400 in PBS containing 0.1% Tween 20 (PBS-T) and 5% milk powder. Subsequently, detection was continued using horseradish peroxidase- (HRP-)coupled goat anti mouse IgG (#401215), goat anti rabbit IgG (#401315) (both from Calbiochem, Darmstadt, Germany), or donkey anti goat IgG (sc-2020) (Santa Cruz Biotechnology, Santa Cruz, CA, USA). If appropriate signals were lacking, electrophoresis and blotting were repeated, and detection was performed using secondary biotinylated goat anti rabbit, horse anti mouse, or horse anti goat IgG (BA-1000, BA-2000, BA-9500) (Vector Laboratories, Burlingame, CA, USA), followed by incubation with avidin and biotinylated HRP from the “Vectastain ABC kit” (Vector Laboratories, Burlingame, CA, USA) both diluted 1:200 in PBS. Membranes were washed with PBS-T after any incuabation with primary or secondary antibodies, or biotin-HRP. Finally, blots were developed with enhanced chemiluminescence (ECL) using ECL Hyperfilm (GE Healthcare, Freiburg, Germany).

### Immunofluorescence

Human prostate specimens, embedded in optimal cutting temperature (OCT) compound, were snap-frozen in liquid nitrogen and kept at -80°C. Sections (8 μm) were cut in a cryostat and collected on Superfrost^®^ microscope slides. Sections were post-fixed in methanol at -20°C and blocked in 1% bovine serum albumin before incubation with primary antibody over night at room temperature. For double labeling, following primary antibodies were used: rabbit anti PAK1 (#2602) (New England Biolabs, Ipswich, MA, USA), goat anti γPAK (sc-7117), rabbit PAK4 (sc-28779), rabbit PAK6 (sc-32857), mouse anti pan-cytokeratin (sc-8018), mouse anti calponin 1/2/3 (sc-136987), mouse anti prostate-specific antigen (PSA) (sc-7316), or mouse anti β-actin antibody (sc-47778), mouse anti tyrosine hydroxylase (sc-374048), mouse anti pan-cytokeratin (sc-8018), and mouse anti calponin 1/2/3 (sc-136987) (if not other stated, all from Santa Cruz Biotechnology, Santa Cruz, CA, USA). Binding sites were visualized using Cy3-conjugated goat anti mouse IgG (AP124C), fluorescein isothiocyanate- (FITC-)conjugated rabbit anti goat IgG) (AP106F) (both from Millipore, Billerica, MA, US, and Cy5-conjugated goat anti rabbit IgG (ab6564) (Abcam, Cambridge, UK). Nuclei were counterstained with 4’,6’-diamidino-2-phenylindole-dihydrochloride (DAPI) (Invitrogen, Camarillo, CA, USA). Immunolabeled sections were analyzed using a laser scanning microscope (Leica SP2, Wetzlar, Germany). Fluorescence was recorded with separate detectors. Control stainings without primary antibodies did not yield any signals.

### Tension measurements

Prostate strips (6 x 3 x 3 mm) were mounted in 10 ml aerated (95% O_2_ and 5% CO_2_) tissue baths (Föhr Medical Instruments, Seeheim, Germany), containing Krebs-Henseleit solution (37°C, pH 7.4). Preparations were stretched to 4.9 mN and left to equilibrate for 45 min. In the initial phase of the equilibration period, spontaneous decreases in tone are usually observed. Therefore, tension was adjusted three times during the equilibration period, until a stable resting tone (4.9 mN) was attained. After the equilibration period, maximum contraction induced by 80 mM KCl was assessed. Subsequently, chambers were washed three times with Krebs-Henseleit solution for a total of 30 min. Cumulative concentration response curves for noradrenaline or phenylephrine, or frequency response curves induced by electric field stimulation (EFS) were created after addition of PAK inhibitors, or dimethylsulfoxide (DMSO) for controls. Application of EFS simulates action potentials, resulting in the release of endogenous neurotransmitters, including norepinephrine. For calculation of agonist- or EFS-induced contractions, tensions were expressed as % of KCl-induced contractions, as this may correct different stromal/epithelial ratios, different smooth muscle content, varying degree of BPH, or any other heterogeneity between prostate samples and patients.

### Cell culture

WPMY-1 cells are an immortalized cell line obtained from nonmalignant human prostate stroma. Cells were obtained from American Type Culture Collection (ATCC; Manassas, VA, USA), and kept in RPMI 1640 (Gibco, Carlsbad, CA, USA) supplemented with 10% fetal calf serum (FCS) and 1% penicillin / streptomycin at 37°C with 5% CO_2_. Before addition of FRAX486, IPA3, or DMSO (for controls) the medium was changed to a FCS-free medium. For Western blot analysis, cells were lyzed using radioimmunoprecipitation assay (RIPA) buffer (Sigma-Aldrich, St. Louis, MO, USA), and removed from flasks after 15 min of incubation on ice. Cell debris was removed by centrifugation (10,000 g, 10 min, 4°C), and different aliquots of supernatants were either subjected to protein determination, or boiled with SDS sample buffer.

### Phalloidin staining

For fluorescence staining with phalloidin, cells were grown on Lab-Tek Chamber slides (Thermo Fisher, Waltham, MA, USA) with inhibitors, or solvent. Staining was performed using 100 μM FITC-labeled phalloidin (Sigma-Aldrich, Munich, Germany), according to the manufacturer’s instruction. Labeled cells were analyzed using a laser scanning microscope (Leica SP2, Wetzlar, Germany).

### Cytotoxicity assay

Cytotoxicity of PAK inhibitors was assessed using the Cell Counting Kit-8 (CCK-8) (Sigma-Aldrich, St. Louis, MO, USA). Cells were grown in 96-well plates (20,000 cells/well) for 24 h, before FRAX486, IPA3, or DMSO were added in indicated concentrations (1, 5, 10 μM). Subsequently, cells were grown for different periods (24, 48, 72 h). Separate controls were performed for each period. At the end of this period, 10 μl of [2-(2-methoxy-4-nitrophenyl)-3-(4-nitrophenyl)-5-(2,4-disulfophenyl)-2H-tetrazolium monosodium salt (WST-8) from CCK-8 was added, and absorbance in each well was measured at 450 nm after incubation for 2 h at 37°C.

### Cell proliferation assay

WPMY-1 cells were plated with a density of 50,000/well on a 16-well chambered coverslip (Thermo Scientific, Waltham, MA, USA). After 24 h, cells were treated with FRAX486 (1, 5, 10 μM), IPA3 (1, 5, 10 μM), or DMSO. After further 24 h, the medium was changed to a 10 mM 5-ethynyl-2’-deoxyuridine (EdU) solution in FCS-free medium containing inhibitors or solvent. 20 h later, cells were fixed with 3.7% formaldehyde. EdU incorporation was determined using the “EdU-Click 555” cell proliferation assay (Baseclick, Tutzing, Germany) according to the manufacturer`s instructions. In this assay, incorporation of EdU into DNA is assessed by detection with fluorescing 5-carboxytetramethylrhodamine (5-TAMRA). Counterstaining of all nuclei was performed with DAPI. Cells were analyzed by fluorescence microscopy (excitation: 546 nm; emission: 479 nm).

### Phosphorylation assessments

Activation of PAK1-3 involves autophosphorylation at threonine 402 or corresponding sites (threonine 423), which may be assessed using site- and phospho-specific antibodies [8, 35–37]. For assessments of agonist-induced PAK phosphorylation, WPMY-1 cells were grown in T75 flasks. After 48 h, medium was changed to FCS-free medium. After 24 h, serum-starved cells were stimulated with endothelin-1 (3 μM) or endothelin-2 (3 μM) for 10 or 20 min, with noradrenaline (100 μM) for 5 or 10 min, or with dihydrotestosterone (100 μM) for 8 or 48 h. Controls without stimulation but identical total exposure time were included in each single experiment and for each agonist. Subsequently, cells were lysed using lysis buffer, and removed from flasks after 15 min of incubation on ice. Cell debris was removed by centrifugation (10,000 g, 10 min, 4°C), and aliquots of supernatants were directly subjected to protein determination. These samples were subjected to Western blot analysis for phospho-PAK, PAK1, or β-actin, which was performed using rabbit anti p-α/β/γPAK (Thr402) (sc-135684), mouse anti αPAK (sc-166887), or mouse anti β-actin antibody (sc-47778) (all from Santa Cruz Biotechnology, Santa Cruz, CA, USA). On each Western blot, samples from the same experiment were compared (agonist vs. controls). Intensities of resulting bands were quantified densitometrically using “Image J” (NIH, Bethesda, Maryland, USA). For semiquantitative calculation, samples without agonists (controls) were set to 100%, and samples with agonists were expressed as % of controls. This normalization is inevitable due to high variation of control values obtained by densitometric quantification (providing “arbitrary units”). These variations mostly result from detection conditions (slight differences of incubation periods, exposure times), and from different adjustments during digitization of blots (required due to varying background or PAK content). These variations of control values may practically not be avoided; therefore, normalization of controls was required. However, groups containg normalized controls should not be subjected to t-tests, so that statistical tests were not applied to quantifications of phosphorylation experiments.

### Drugs and nomenclature

6-(2,4-Dichlorophenyl)-8-ethyl-2-[[3-fluoro-4-(1-piperazinyl)phenyl]amino]pyrido[2,3-d]pyrimidin-7(8*H*)-one (FRAX486) and 1,1'-dithiodi-2-naphthtol (IPA3) are structurally unrelated inhibitors of PAKs. Stock solutions (10 mM) were prepared with DMSO, and kept at -20°C until use. Phenylephrine ((*R*)-3-[-1-hydroxy-2-(methylamino)ethyl]phenol) is a selective agonist for α_1_-adrenoceptors. Aqueous stock solutions of phenylephrine and norepinephrine (10 mM) were freshly prepared before each experiment. FRAX486 and IPA3 were obtained from Tocris (Bristol, UK), phenylephrine and norepinephrine were obtained from Sigma (Munich, Germany).

### Statistical analysis

Data are presented as means ± standard error of the mean (SEM) with the indicated number (n) of experiments. Two-tailed student *t* test was used for paired or unpaired observations. *P* values <0.05 were considered statistically significant.

## Results

### Detection of PAK isoforms in prostate tissues and WPMY-1 cells

**Fig 1:**
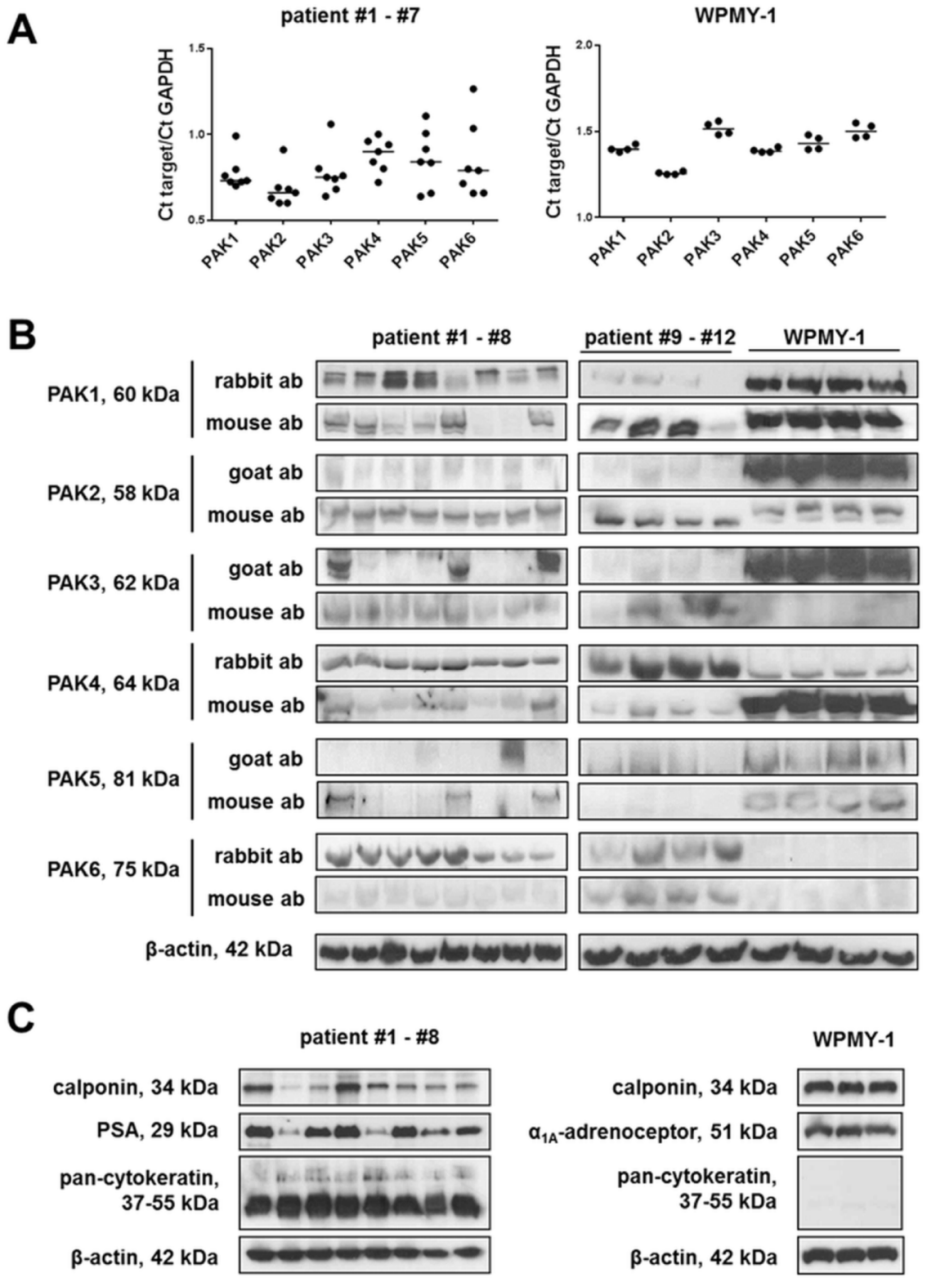
Detection of PAK isoforms and markers in human prostate tissue, and in WPMY-1 cells. Detection was performed by RT-PCR **(A)**, or Western blot analyses **(B, C)**. Shown are ratios of Ct values (target/GAPDH) for each sample (from n = 7 patients, or from WPMY-1 cells from 4 experiments) **(A)**, or bands for all investigated samples (from n = 12 patients, or WPMY-1 cells from 4 experiments **(B)**. In **(B) and (C)**, indicated molecular weights are the expected sizes of proteins. Detection of each PAK isoform in **(B)** was performed with two different antibodies for each isoform (mouse, and rabbit or goat) (ab, antibody). Western blots included calponin as a marker for smooth muscle cells, pan-cytokeratin as a marker of endothelial cells (glands), prostate-specific antigen (PSA) as a marker for hyperplasia, and α_1A_-adrenoceptors as an important feature of prostate smooth muscle cells.

The PAK family comprises PAK1 (syn. α-PAK), PAK2 (syn. γ-PAK), PAK3 (syn. β-PAK), PAK4, PAK5 (syn. PAK7), and PAK 6 (syn. PAK5) [10, 11]. We detected mRNA and protein of different isoforms in periurethral tissues from hyperplastic human prostates, and in cultured WPMY-1 cells. Results from all analyses including RT-PCR, Western blot, and immunofluorescence staining are summarized in Table 1. In tissues and cells, mRNA for all PAK isoforms was detectable (Fig 1A, Table 1).

**Table 1:** Summarized results from detection of mRNA and protein of different PAK isoforms. Detections were performed by RT-PCR, Western blot analysis, and immunofluorescence staining. Detection by Western blot was performed with to different.

	Prostate tissues			WPMY-1	
	RT-PCR	Western blot	IF	RT-PCR	Western blot
**PAK1**	detectable in each sample; content similar in all samples (apart from one runaway)	detectable with rabbit or mouse ab, and in almost each sample; but content varies considerably between samples	colocalization with calponin; colocalization with TH	detectable; content: WPMY-1 > prostate	stronlgy detectable with rabbit or mouse ab; content: WPMY-1 > prostate
**PAK2**	detectable in each sample; content similar in all samples (apart from one runaway)	detectable using mouse ab, suggesting similar content in all samples; only hardly detectable in all samples using goat ab	almost no colocalization with calponin; colocalization with TH	detectable; content: WPMY-1 > prostate	stronlgy detectable with goat ab, still good detectable with mouse ab; content: WPMY-1 > or ≥ prostate
**PAK3**	detectable in each sample; content similar in all samples (apart from one runaway)	strong bands in 3/8 samples, but completely undetectable in 5/8 samples using goat ab; only faint bands using mouse ab	n. a.	detectable; content: WPMY-1 > prostate	stronlgy detectable with goat ab, undetectable with mouse ab; content: WPMY-1 > prostate with goat ab
**PAK4**	detectable in each sample; content varies between samples	Clearly detectable in each sample using rabbit ab, suggesting similar content in all samples; only hardly detectable in most samples using goat ab	almost no colocalization with calponin; colocalization with TH	detectable; content: WPMY-1 > prostate	stronlgy detectable with mouse ab, weakly detectable with rabbit ab; no reliable conclusion about content possible
**PAK5**	detectable in each sample; content varies strongly levels	strong bands in 3/8 samples, but completely undetectable in 5/8 samples using mouse ab; clear bands in 2/8 samples using goat ab	n. a.	detectable; content: WPMY-1 > prostate	bands blurred but detectable using mouse or goat ab; content: WPMY-1 > prostate
**PAK6**	detectable in each sample; content varies strongly levels	Clearly detectable in each sample using rabbit ab, suggesting groups with either high or low content; almost undetectable using mouse ab	no colocalization with calponin; colocalization with TH	detectable; content: WPMY-1 > prostate	undetectable using rabbit or mouse antibody; content: prostate > WPMY-1

Western blot analyses were performed using isoform-specific PAK antibodies, and repeated using two different antibodies for each isoform. For evaluation, only bands with sizes matching the corresponding molecular weight of the isoform, or regions covering these sizes were considered. Diverging and additional bands occurred with some antibodies, but were not regarded for conclusions (S1 Fig).

In Western blot analysis of prostate tissues, the best detectable isoform was PAK1 (Fig 1B). Bands matching the expected molecular weight of PAK1 were detected using two different antibodies, and in most samples being included to this analysis (Fig 1B). Despite detection in nearly all samples, varying intensity of bands suggested variations of PAK1 content between different patients (Fig 1B). Variation was confirmed by both applied antibodies (Fig 1B). PAK3 and -5 were detectable with strong bands in only few prostate samples, while they were completely undetectable or only very faintly detectable in most samples (Fig 1B). PAK2, -4, and -6 were detectable with almost constant intensity with one antibody, but only faintly detectable or undetectable with another antibody (Fig 1B). Together, this suggests a constitutive expression at least of PAK1, and possibly of PAK2, -4, and -6, while expression of PAK3 and -5 may be inducible (Table 1).

Similar to prostate tissues, PAK1 was the isoform, which was best detectable from all isoforms in Western blot analysis of WPMY-1 cells, using two different antibodies (Fig 1B). In contrast, PAK6 was completely undetectable even using two different antibodies (Fig 1B). Other isoforms were strongly detectable with one antibody, but only faintly detectable (PAK2, -4) or undetectable (PAK3) with another, while blurred bands for PAK5 appeared after detection with two different antibodies (Fig 1B). Comparison with prostate tissues suggested that at least the expression of PAK1 and PAK5 in WPMY-1 cells may be higher than in prostate tissues (Fig 1B, Table 1), what was confirmed by RT-PCR (Fig 1A, Table 1).

**Fig 2:**
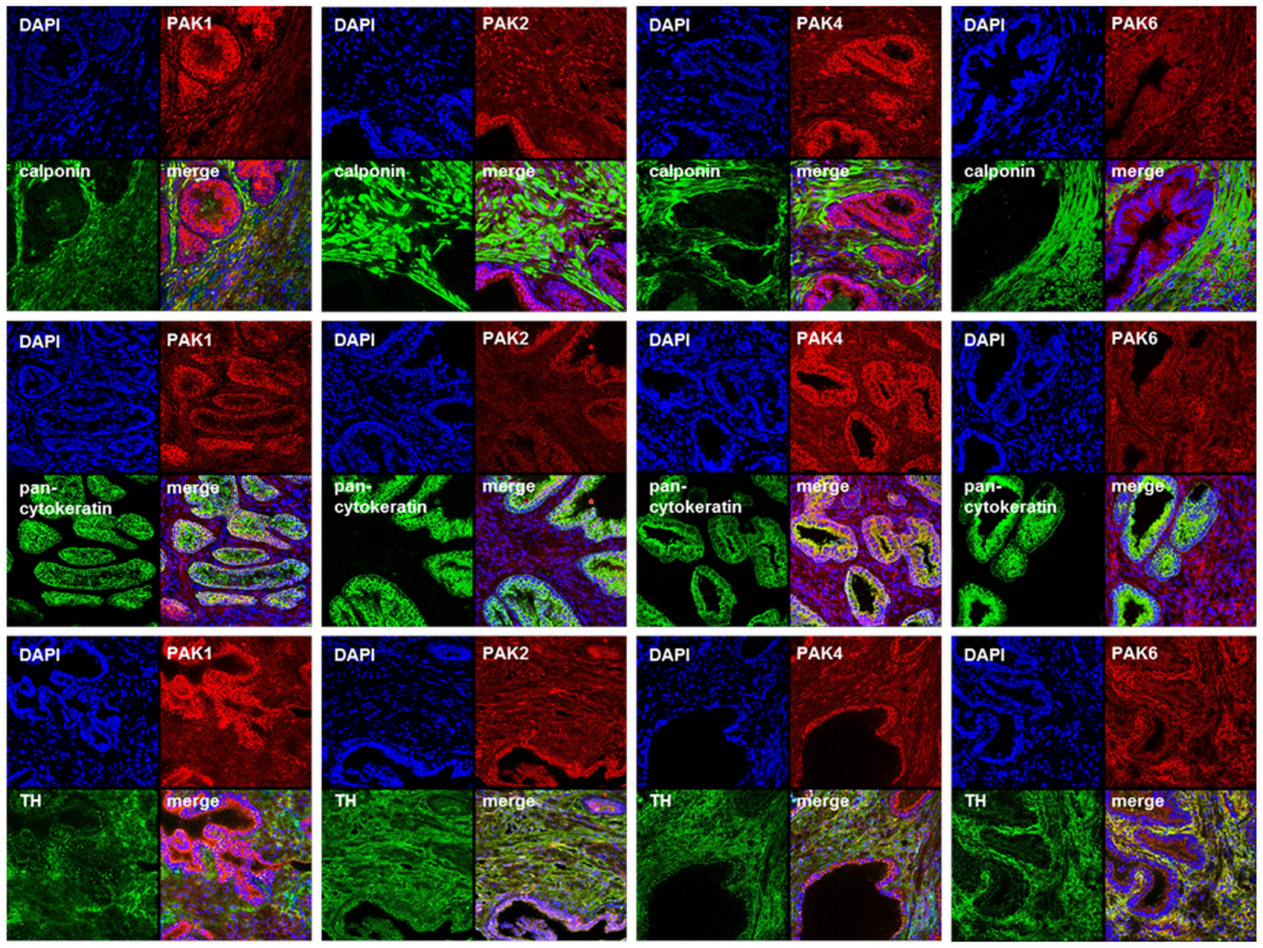
Immunofluorescence stainings of human prostate tissues. Sections were double labeled for different PAK isoforms, together with calponin (marker for smooth muscle cells), pan-cytokeratin (marker for epithelial cells of glands), or tyrosine hydroxylase (TH, marker for catecholaminergic nerves). Yellow color in merged pictures may indicate colocalization of targets. Shown are representative stainings from series with tissues from n = 5 patients for each combination.

In fluorescence stainings, sections of prostate tissues were double labeled for markers (calponin for smooth muscle cells, or pan-cytokeratin for epithelial cells of glands) and for those PAK isoforms, for which a constitutive expression may be assumed (PAK1, -2, -4, and -6) (Table 1). Isoform-specific PAK antibodies used for fluorescence labelings provided exclusively (PAK1, -2, -6) or prevailingly (PAK4) single bands with sizes of the expected molecular weights in Western blot analyses (S1 Fig). Stainings suggested that PAK1 may be the prevailing isoform with constitutive expression in smooth muscle cells, whereas PAK1, PAK2, PAK4, and PAK6 may be found in catecholaminergic nerves (Fig 2).

BPH to different degree is considered typical for patients with prostate cancer, and is reflected by varying levels of prostate specific antigen (PSA) [38]. Hyperplasia in prostate samples was confirmed by varying content of PSA, calponin (smooth muscle marker) and pan-zytokeratin (epithelial marker, staining glandular cells) (Fig 1C). In WPMY-1 cells, α_1A_-adrenoceptors and the smooth muscle marker calponin, two important features of prostate smooth muscle cells were detectable, while pan-cytokeratin, a characteristic marker for epithelial, glandular cells, was lacking (Fig 1C).

### Inhibition of prostate smooth muscle contraction by PAK inhibitors

**Fig 3:**
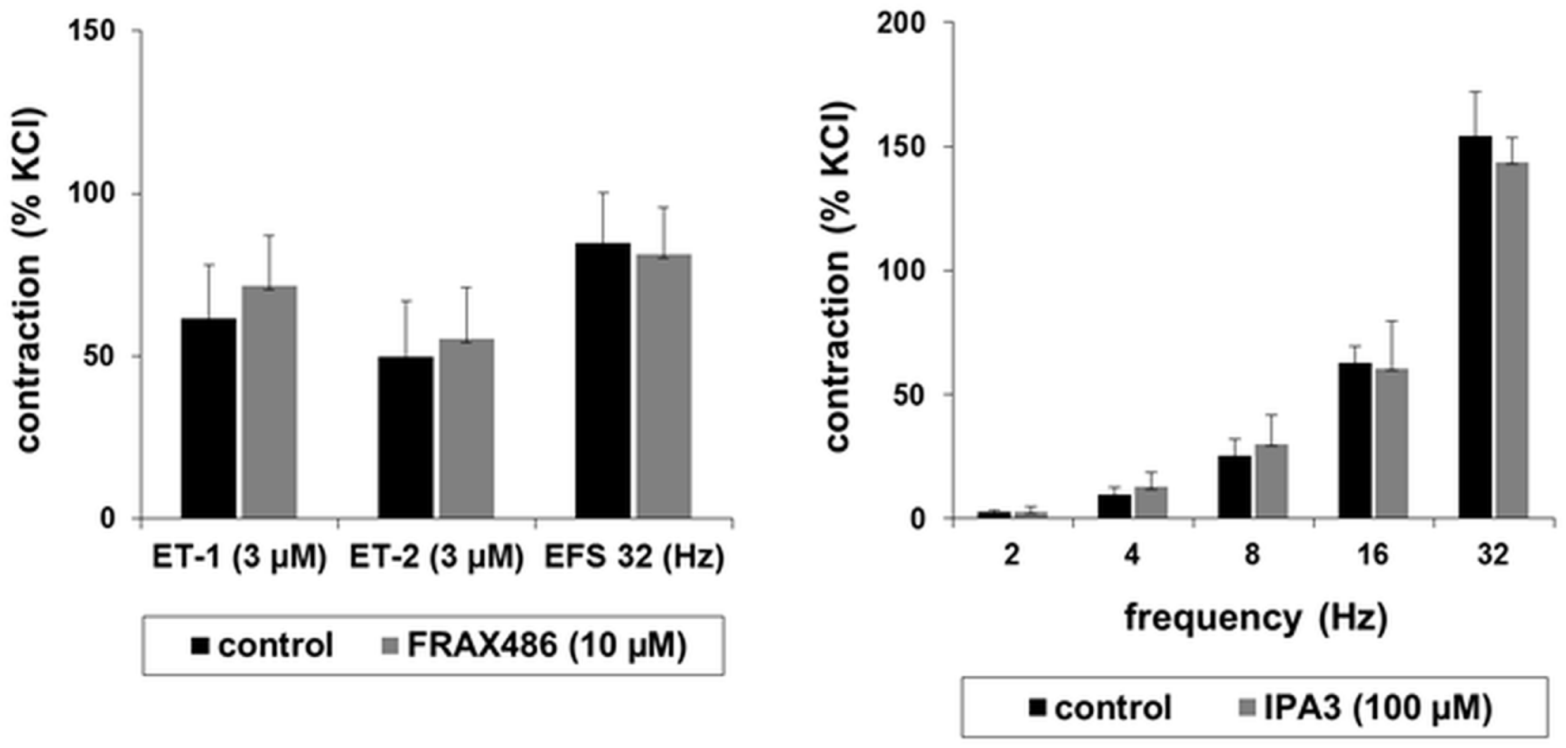
Effects of FRAX486 and IPA3 in low concentrations on contraction of human prostate strips. Contractions of isolated human prostate strips were induced as indicated, after addition of 10 μM FRAX48, 100 μM IPA3, or DMSO (control). Tensions have been expressed as % of contraction by highmolar KCl, being assessed before application of inhibitors or solvent. This normalization allows comparisons despite different conditions of tissues or patients, e. g. due to varying degree of BPH, different content of smooth muscle, or any other heterogeneity (compare Fig 1C). Shown are means ± SEM from experiments with tissues from n = 5 (endothelin-1, EFS, each with FRAX486), n = 4 (endothelin-2), or n = 3 (EFS with IPA3) patients for each group.

**Fig 4:**
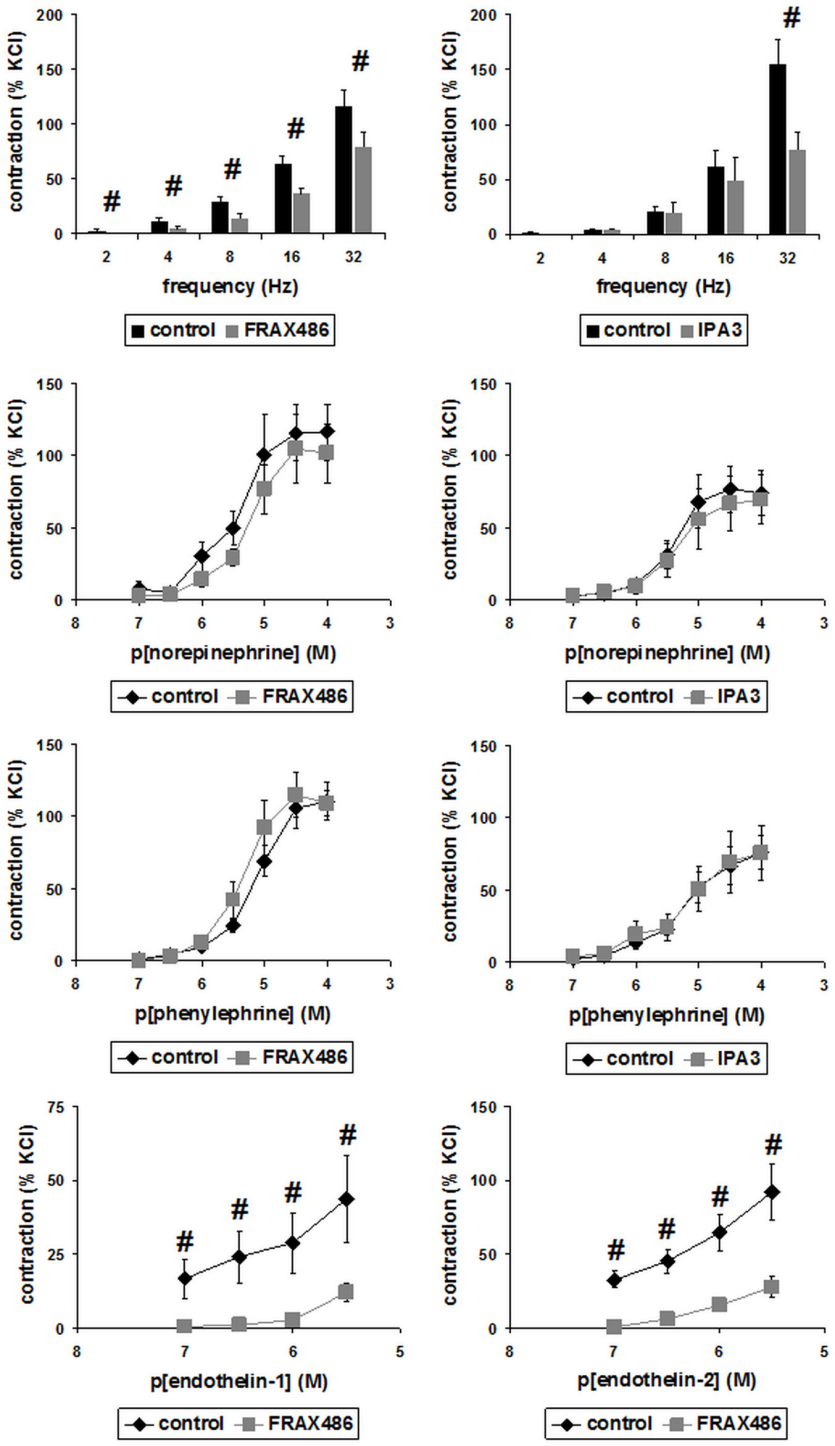
Effects of FRAX486 and IPA3 in high concentrations on contraction of human prostate strips. Contractions of isolated human prostate strips were induced as indicated, after addition of FRAX48 (30 μM), IPA3 (300 μM), or DMSO (control). Tensions have been expressed as % of contraction by highmolar KCl, being assessed before application of inhibitors or solvent. This normalization allows comparisons despite different conditions of tissues or patients, e. g. due to varying degree of BPH, different content of smooth muscle, or any other heterogeneity (compare Fig 1C). Shown are means ± SEM from experiments with tissues from n = 5–8 patients for each group (# p<0.05).

PAKs may be involved in smooth muscle contraction of the airways, cardiovascular system, and gastrointestinal tract [13–21]. This prompted us to examine effects of two different, small molecule PAK inhibitors, FRAX486 and IPA3 on contraction of human prostate smooth muscle. Selectivity of both inhibitors is highest for group I PAKs (i. e., PAK1-3), with IC_50_ values below or around the low micromolar range in in vitro kinase assays [36, 39–41]. In organ bath experiments, contractions of prostate strips were induced frequency- or concentration-dependently by electric field stimulation (EFS), noradrenaline, the α_1_-adrenoceptor-selective agonist phenylephrine, endothelin-1, or endothelin-2 (Figs 3 and 4). EFS causes neuronal action potentials, leading to contraction by release of endogenous neurotransmitters [42, 43]. No inhibition of EFS-induced contractions was observed at lower inhibitor concentrations, i. e. using 10 μM of FRAX486 or 100 μM of IPA3 (Fig 3). EFS-induced contractions were significantly inhibited by 30 μM of FRAX486 at all frequencies (Fig 4). Similarly, EFS-induced contractions were inhibited by 300 μM of IPA3, which was significant at the highest frequency (32 Hz) (Fig 4). Tensions after significant inhibition ranged around 50% of tensions in controls.

In a concentration of 10 μM, FRAX486 did not inhibit endothelin-induced contractions (Fig 3). Noradrenaline- and phenylephrine-induced contractions were not altered by 30 μM of FRAX486 or 300 μM of IPA3 (Fig 4). Endothelin-1- and -2-induced contractions were significantly inhibited by 30 μM of FRAX486 at each endothelin concentraction (Fig 4). Endothelin-induced tensions after significant inhibition were lower than 50% of tensions in controls.

### Regression of actin organization by PAK inhibitors in WPMY-1 cells

**Fig 5:**
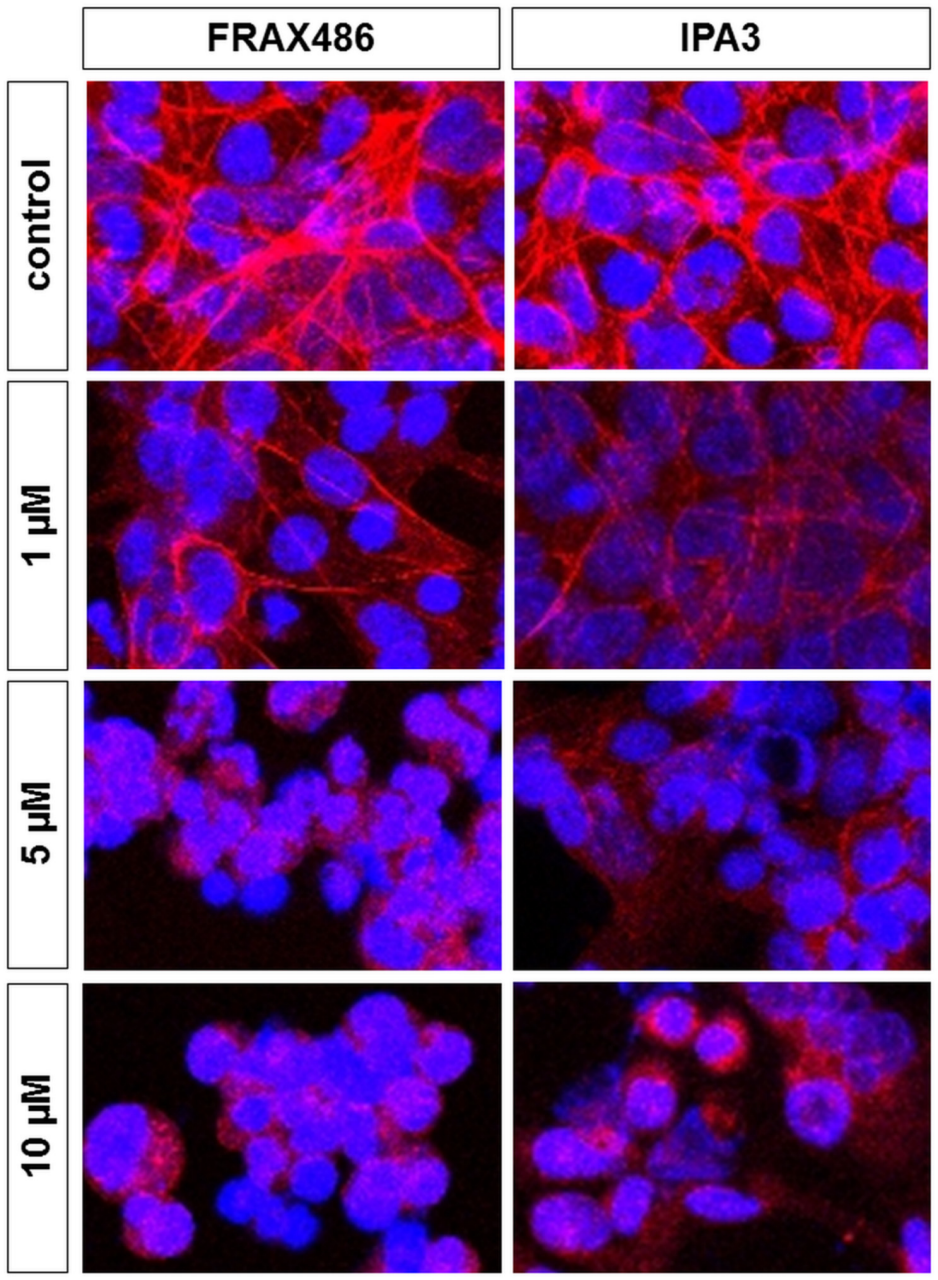
Effects of FRAX486 and IPA3 on actin organization in WPMY-1 cells. Actin filaments were visualized by staining with FITC-coupled phalloidin, after incubation of WPMY-1 cells with DMSO, FRAX486 (1–10 μM), or IPA3 (1–10 μM) for 24 h. Shown are representative images from series with 5 independent experiments, with similar results.

It has been repeatedly reported that PAKs are important regulators of actin organization, so that they may be critical for actin-dependent functions including contraction or proliferation, among others [44–47]. Therefore, we next examined effects of PAK inhibitors on actin organization cultured WPMY-1 cells. FRAX486 and IPA3 (both 1–10 μM, 24 h) caused concentration-dependent degeneration of actin filaments. Actin filaments in solvent-treated control cells were arranged to bundles, forming long and thin protrusions, with elongations from adjacent cells overlapping each other (Fig 5). FRAX486 or IPA3 in concentrations of 1 μM caused partial loss of actin organization, including regressing degree of actin polymerization and degeneration of protrusions (Fig 5). FRAX486 and IPA3 in concentrations of 5 or 10 μM caused complete breakdown of filament organization, resulting in a rounded cell shape without protrusions (Fig 5).

### Cytotoxicity of PAK inhibitors in WPMY-1 cells

**Fig 6:**
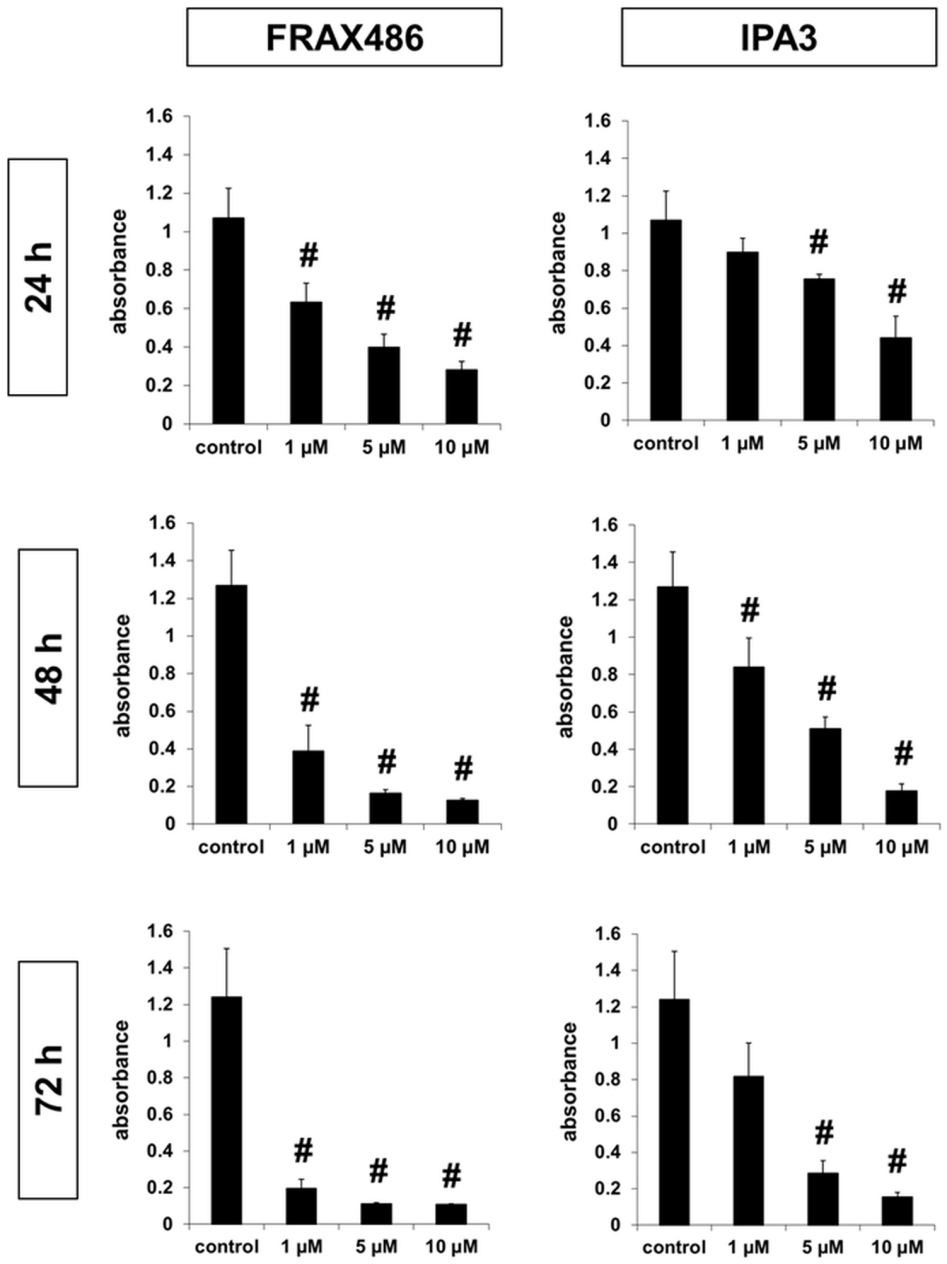
Cytotoxicity of FRAX486 and IPA3 in WPMY-1 cells. Survival of WPMY-1 cells was assessed using CCK-8 assay, after incubation with DMSO, FRAX486 (1–10 μM), or IPA3 (1–10 μM) for 24–72 h. Shown are means ±SEM from series with 5 independent experiments for each setting (# p<0.05 vs. control).

PAKs are critical for cellular survival, which has been mostly investigated in cancer cells [48–50]. Effects of PAK inhibitors on cellular survival may be crucial in vitro, but also for any application in vivo. Therefore, we assessed cytotoxicity of PAK inhibitors in WPMY-1 cells. Cytotoxicity of FRAX486 and IPA3 in WPMY-1 cells turned out to be concentration- and time-dependent. Survival ranged between 55–82% after application of 1 μM FRAX486 for 24 h, or of 1 μM IPA3 for 24–72 h (Fig 6). Higher concentrations and longer application gradually reduced survival: after application of 5 or 10 μM FRAX486 for 48–72 h, or of 10 μM IPA3 for 48–72 h, survival ranged between 1–5% (Fig 6).

### Inhibition of WPMY-1 cell proliferation by PAK inhibitors

**Fig 7:**
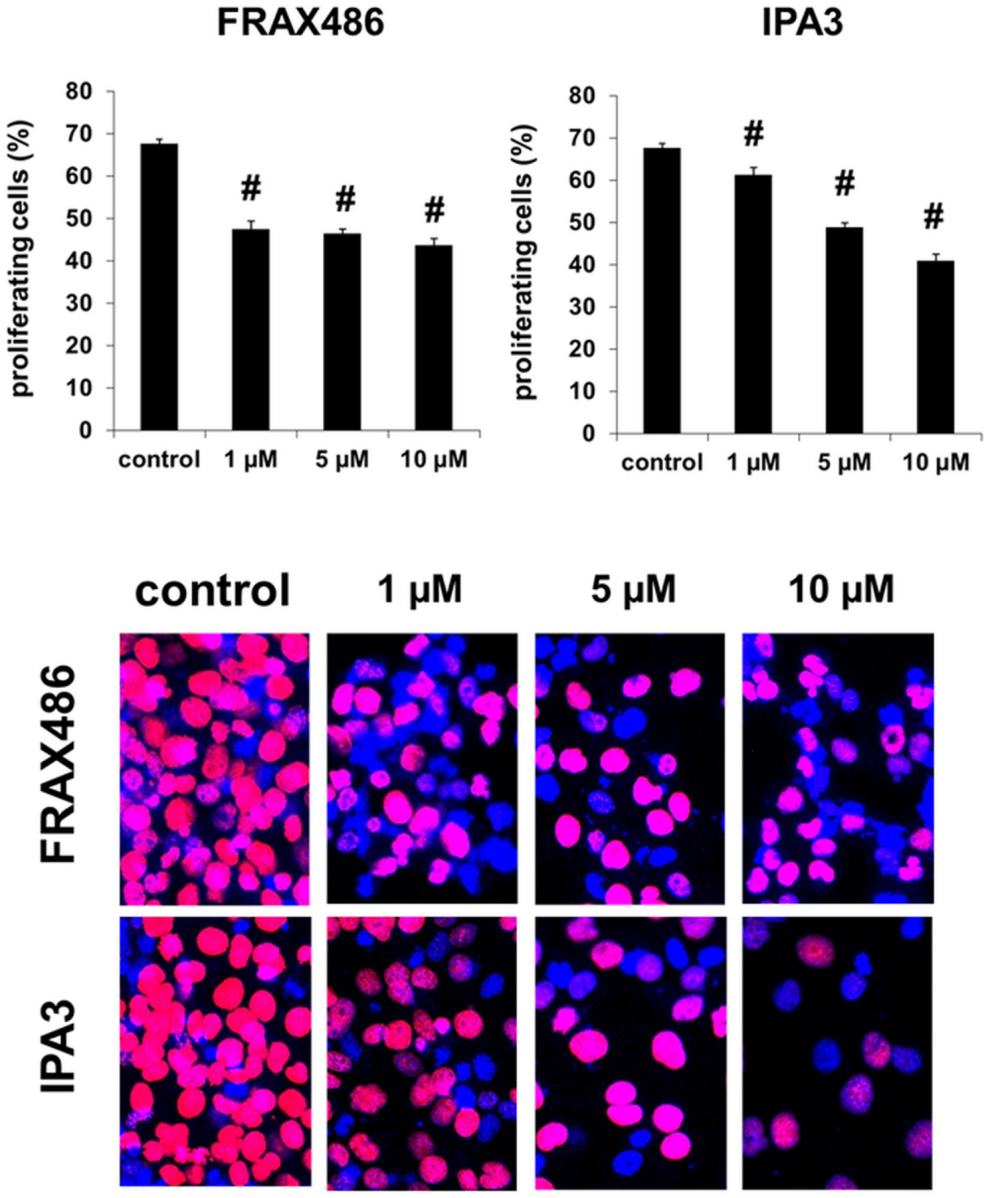
Effects of FRAX486 and IPA3 on proliferation of WPMY-1 cells. Proliferation rate was assessed by EdU assay after incubation with DMSO (control), FRAX486 (1–10 μM), or IPA3 (1–10 μM) for 24 h. The number of cells showing proliferation (= red nuclei) was referred to the total number of cells (= red + blue nuclei), to correct for reduced number of cells after longer incubation periods (compare with Fig 5). Shown are representative images and means ±SEM, from series with 8 independent samples for each setting (# p<0.05 vs. control).

PAKs promote proliferation in various cell types, including airway and vascular smooth muscle cells [22–25]. As any effect on proliferation of prostate cells may be interesting for application in BPH, where hyperplastic growth induces symptoms, we examined effects of PAK inhibitors on proliferation of WPMY-1 cells. FRAX486 and IPA3 significantly reduced the relative proliferation rate in the remaining populations of WPMY-1 cells (Fig 7). While 68% of solvent-treated (24 h) cells showed proliferation, proliferation rate after application of FRAX486 (1–10 μM, 24 h) ranged around 45%, or between 41–61% after application of IPA3 (1–10 μM, 24 h) (Fig 7).

### Agonist-induced PAK phosphorylation in WPMY-1 cells

**Fig 8:**
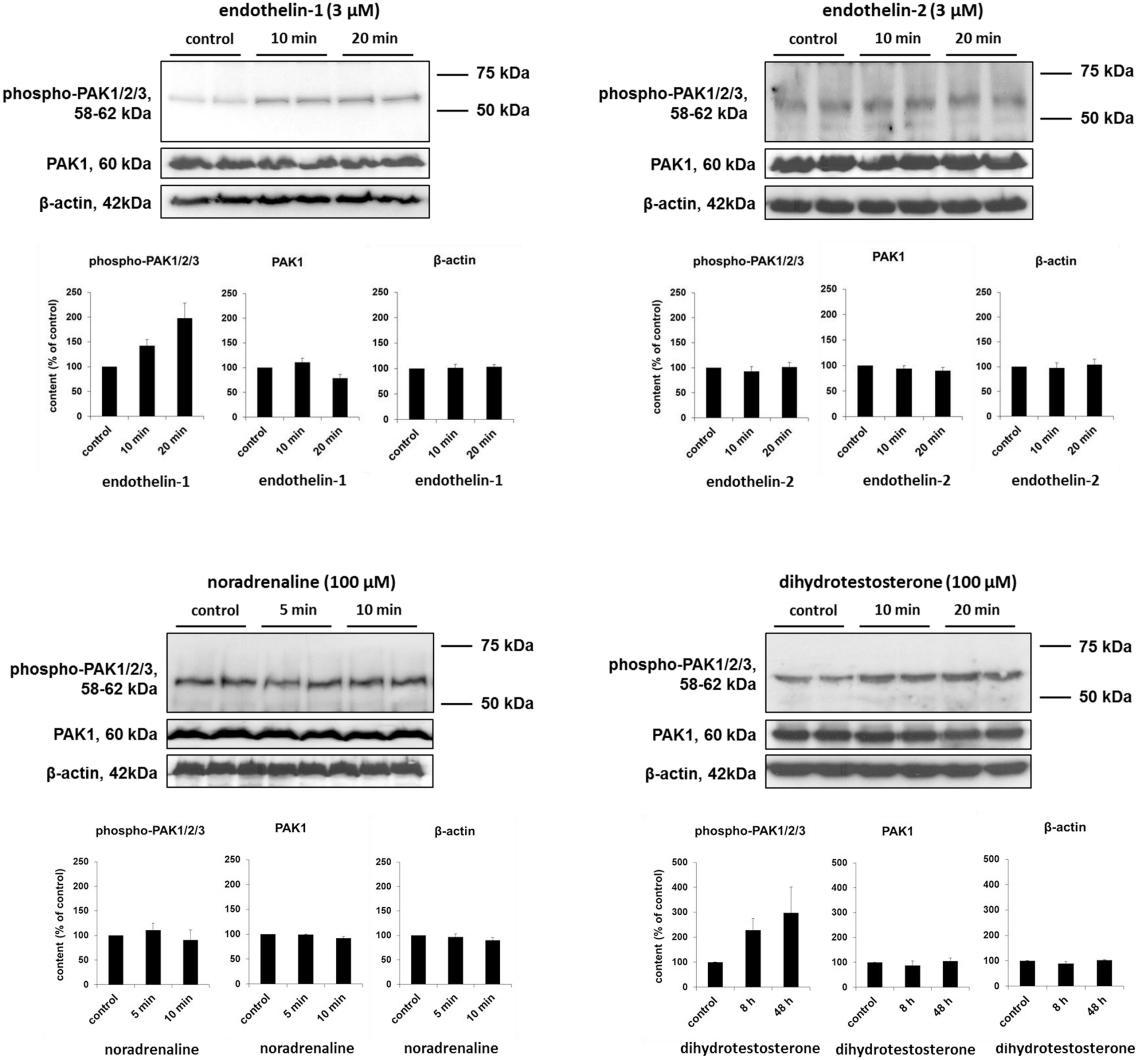
Effects of agonist stimulation on PAK phosphorylation in WPMY-1 cells. WPMY-1 cells were stimulated with agonists as indicated, or remained without stimulation (controls). Subsequently, Western blot analyses were performed for phospho-PAK (using a phospho-specific antibody recognizing PAK1-3 phosphorylated at threonine 402 or corresponding sites), PAK1, and β-actin. Shown are representative Western blots and densitometric quantification of all experiments (series with n = 5 independent experiments for each agonist) (means ±SEM).

Finally, we assessed whether PAKs may be activated by noradrenaline, endothelins, or dihydrotestosterone in WPMY-1 cells. Activation of group I PAKs includes phosphorylation at common positions, which may be investigated using phospho-specific antibodies [35–37]. Stimulation of WPMY-1 cells with endothelin-1 (3 μM) for 10 or 20 min increased the content of phosphorylated group I PAKs (i. e., PAK1-3), while the content of PAK1 and β-actin was similar in stimulated and control cells (Fig 8). Similarly, increased phospho-PAK levels were observed after stimulation with dihydrotestosterone (100 μM) for 8 or 48 h, while the content of PAK1 and β-actin remained constant during stimulation (Fig 8). Maximum agonist-induced phospho-PAK content was 198 ± 30% from controls after stimulation with endothelin-1 (20 min), or 297 ± 104% from controls after stimulation with dihydrotestosterone (48 h). Stimulation with endothelin-2 (3 μM) for 10 or 20 min, or with noradrenaline (100 μM) for 5 or 10 min did not change the content of phospho-PAK, PAK1, or β-actin (Fig 8).

## Discussion

**Fig 9:**
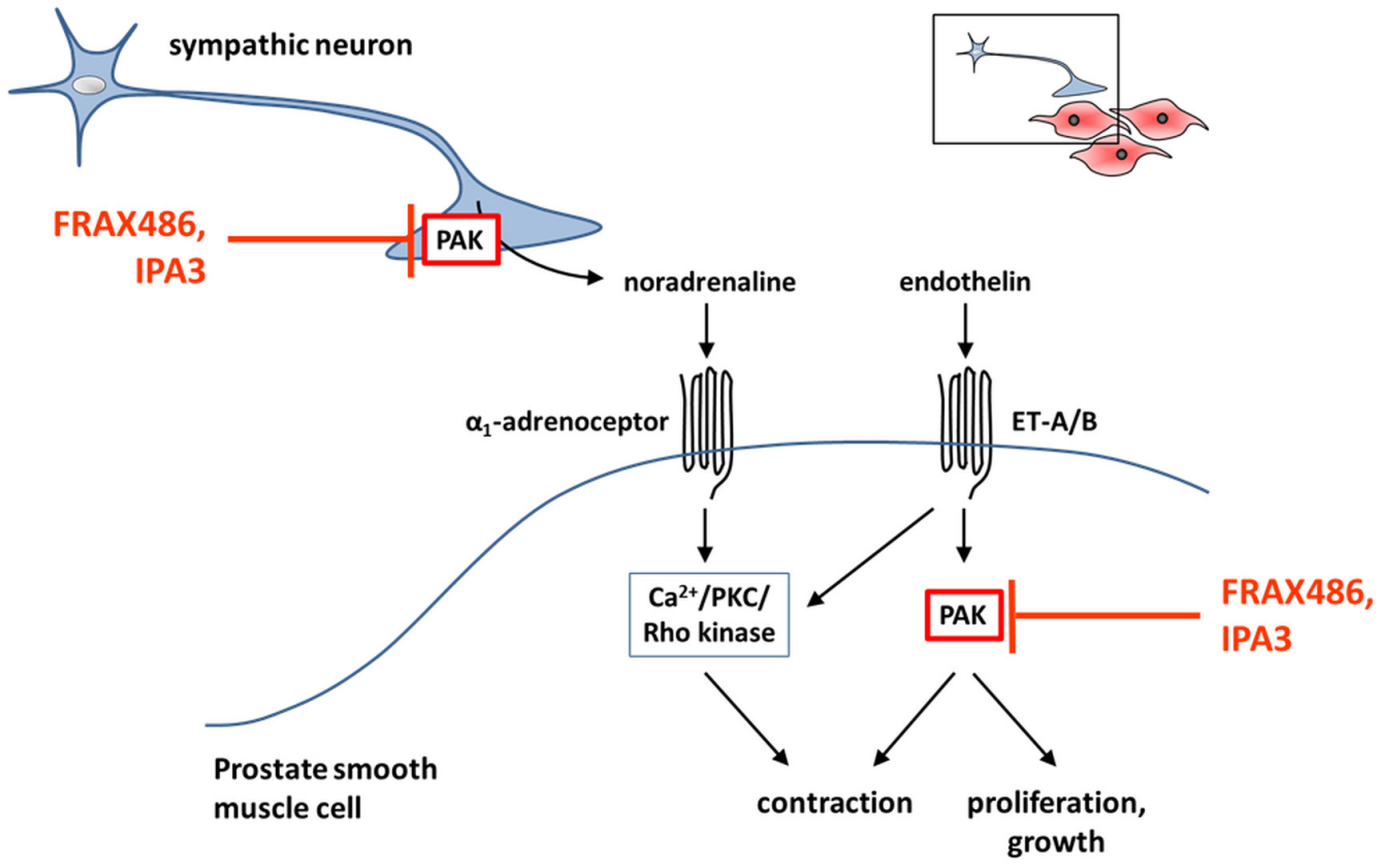
Assumed PAK signaling in the hyperplastic human prostate, and intervention by PAK-specific small molecule inhibitors. The presented model is based on findings of this study. At least two different PAK functions may critically determine adrenergic and endothelin-mediated contraction of prostate smooth muscle. First, PAK promotes the release of noradrenaline during sympathic neurotransmission to smooth muscle cells, followed by contraction by activation of postsynaptic α_1_-adrenoceptors. Consequently, PAK inhibitors inhibited EFS-induced contractions of prostate strips. Secondly, PAK in smooth muscle cells mediates contraction, where PAK is selectively activated by endothelin receptors (ET-A/B), but not by α_1_-adrenceptors. Possibly, PAK mediates the contractile signal by ET-A/B, in parallel to established intracellular pathways, which include Ca^2+^, protein kinase C (PKC), and Rho kinase. This might explain, why PAK inhibitors inhibited endothelin-induced contractions of prostate strips, but not contractions by α_1_-agonists. Besides its role for smooth muscle tone, PAK inhibition reduced proliferation of stromal cells, suggesting a role for prostate growth.

Our findings show that it may be principally possible to target smooth muscle contraction and growth in the prostate at once, by single compounds. Both processes are critical for etiology and therapy of voiding symptoms in patients with BPH, but combination therapies are still required to reduce both at once [2–4]. Certainly, available medical options may improve the situation of many patients. Altogether, however, insufficient efficacy, disappointing responder rates, and high discontinuation rates provide clear limitations of current pharmacotherapy [2–4, 51–56]. Although drugs concurrently addressing smooth muscle contraction and hyperplastic growth in the prostate may be attractive putative candidates for treatment of male LUTS, this possibility has been rarely considered. Here, we examined effects of PAK inhibitors on prostate smooth muscle contraction, and growth of stromal cells. Based on these observations in vitro, it may be expected that PAK inhibitors induce urodynamic effects in vivo. While available compounds for medical treatment of male LUTS either address (α_1_-adrenergic) contraction, *or* prostate size, PAK inhibitors were capable to affect adrenergic *plus* non-adrenergic contraction, *and* stromal cell growth at once in our study (Fig 9). We are aware that any efficacy of PAK inhibitors for improvement of LUTS needs to be confirmed in vivo, and that applications may be limited by side-effects. FRAX486 has been developed recently, and its application for several days was tolerated in mice [40, 57].

We used tissues from the periurethral zone, taken from prostates after radical prostatectomy for prostate cancer. We consider these samples as non-malignant and mostly hyperplastic for two reasons. First, samples were taken from the periurethral zone, while the great majority of tumors in prostate cancer is located to the peripheral zone [31, 32]. Consequently, macroscopical inspection of our radicals by pathologists before sampling never revealed tumors in the periurehtral zone. Secondly, most patients undergoing radical prostatectomy show BPH; it has been estimated that 80% of patients with prostate cancer show BPH [33, 34]. This is in line with the overall high prevalence of BPH, which increases with age. Thus, around 80% of men with an age of 80 years or older have BPH, while the average age of our patients undergoing radical prostatectomy ranges between 61–68 years [7, 58–60]. We confirmed BPH by detection of PSA in Western blot analysis: as the content of PSA increases with the degree of BPH, varying content of PSA in our samples reflects BPH with varying degree [38].

Our findings suggest divergent PAK-dependent regulation of endothelin- and α_1_-adrenoceptor-mediated contractions. As FRAX486 and IPA3 inhibited EFS-induced, but not noradrenaline- or phenylephrine-induced contractions of prostate strips at least at higher concentrations, it may be assumed that PAKs might be critical for adrenergic neurotransmission in the hyperplastic human prostate, possibly by regulation of noradrenaline release. Double labeling of prostate sections with TH suggested expression of different PAK isoforms in catecholaminergic nerves of hyperplastic prostate tissues. In fact, expression of all PAK isoforms elsewhere in the nervous system is well known, and investigation of PAK regulation was often related to neuronal functions [10]. However, we cannot estimate which isoforms are involved in adrenergic neurotransmission in the prostate. This resembles the situation in many organs and cell types, where PAK functions are often poorly understood at isoform level [39, 61].

As PAK1 may be the prevailing isoform in prostate smooth muscle cells, inhibition of PAK1 may be responsible for inhibition of endothelin-induced contractions by FRAX486. Obviously, PAK1 is capable to distinguish endothelin receptors from α_1_-adrenoceptors in smooth muscle cells, so that endothelin- but not α_1_-adrenoceptor-induced contraction is promoted by PAK1. Our phosphorylation experiments in WPMY-1 cells suggest that endothelin receptors, but not α_1_-adrenoceptors induce PAK activation. In fact, it has been previously suggested that PAKs may be involved in endothelin receptor-induced signaling [62]. This may explain selective effects of FRAX486 in our contraction experiments. We conclude that PAKs may promote prostate smooth muscle contraction by adrenergic neurotransmission, and by intracellular signaling through endothelin-1-, but not of α_1_-adrenoceptors. This model is summarized in Fig 9. Notably, endothelin-induced force generation was of similar range than α_1_-adrenergic contractions. Such contributions of non-adrenergic mediators to prostate smooth muscle tone may account for limitations of α_1_-blockers [2]. Consequently, it has been proposed that future therapies with higher efficacy than α_1_-blockers have to interfer with adrenergic and non-adrenergic contractions [63].

To the best of our knowledge, our study is the first demonstrating inhibition of smooth muscle contraction by small molecule PAK inhibitors using any intact tissues. Nevertheless, it has been repeatedly proposed that PAKs are important regulators or mediators of smooth muscle contraction [13–16, 18–21, 64–68]. Such studies were based on application of antisense techniques in airway, vascular, or gastrointestinal tissues, or on investigation of cytoskeletal regulation in cell culture models. Different mechanisms have been suggested by which PAKs may promote smooth muscle contraction. It has been assumed that PAKs cooperate with Rac GTPases to promote smooth muscle contraction [64, 67, 69, 70]. In fact, Rac GTPases may be involved in smooth muscle contraction of the prostate [8]. However, PAK-mediated contraction in the prostate seems to be independent from Rac, because interactions between Rac and the putative Rac-binding domain of PAK were not observed in human prostate tissues [8]. Several models proposed PAK-mediated phosphorylation of myosin light chains for smooth muscle contraction [13, 68]. Other models of PAK function included the participation of PAKs in adhesome formation, following activation of the GTPase RhoA by contractile agonists and resulting in polymerization of actin [19, 21]. Actin polymerization is indispensable for smooth muscle contraction, and turned out to be susceptible to Rac inhibitors in WPMY-1 cells [8]. At present, we cannot explain, why we observed deorganization of actin filaments by PAK inhibitors, although α_1_-adrenoceptor-agonist contractions were not inhibited. However, this finding may be in line with a previous study, where IPA3 inhibited actin polymerization, without blunting thromboxane-induced contractions [71]. Together, this suggests that the function of PAK for smooth muscle contraction may differ between organs.

Our Western blot analyses suggested that PAK1 may be a prevailing group I PAK with constitutive expression in human prostate tissue. In fact, bands matching the molecular weight for this isoform were observed with most samples, although with varying intensity. Bands for PAK3 and PAK5 were observed in some, but not all samples, pointing to an expression pattern resembling an on-off principle. This may be explained by two different mechanisms. First, these PAK isoforms are generally not expressed (or at low levels only), but expression can be induced by (unkown) stimuli. Second, these PAK isoforms are generally expressed, but under certain circumstances (in response to unkown stimuli) they are degraded (= regulation at expression level). Regardless from the underlying mechanism, this shows that PAK3 and -5 may be inducible or subject of regulation. Therefore, it should be excluded that PAK3 or -5 account for effects of FRAX486 or IPA3 in prostate tissues.

In WPMY-1 cells, effects of FRAX486 on actin organization, survival, and proliferation occurred already at concentrations of 1–5 μM. In these concentrations, full inhibition of PAK1-3 may be expected, while PAK4 may be inhibited only partially [40]. In vitro kinase assays using pure enzymes revealed IC_50_ values for FRAX486 between 10–100 nM for PAK1-3, while the IC_50_ of 779 nM for PAK4 was just below the micromolar range [40]. It has to be kept in mind, that any IC_50_ in in vitro kinase assays may be considerably lower than EC_50_ values in vivo, or in cultured cells and intact tissues. For FRAX486, an EC_50_ value of 500 nM has been reported from cells (5-50fold higher than IC_50_), so that full effects (“EC_100_”) may be expected in the micromolar range [57]. It appears naturally, that effective concentrations in tissues may be again higher than in cell culture, due to connective tissue, extracellular matrix components, or small interstitial spaces, which may limit accessability of inhibitors to targets in tissues but not in cell culture. At least for FRAX486, increasing the concentrations from 1 μM up to 10 μM did not further increase the effects in WPMY-1 cells, what may suggest that there are no effects from unspecific kinase inhibition (i. e., from non-p21-activated kinases) at higher concentrations. Similarly, we assume that effects of IPA3 in WPMY-1 cells were caused by inhibition of group I PAKs (i. e., PAK1-3), as IPA3 is specific for PAK1-3 within a low micromolar range, but does not inhibit more distantly related PAKs (PAK4-6) or other kinases [36, 39, 41]. Finally, our observation that effective concentrations in prostate tissues were higher than in WPMY-1 cells (i. e., 10 μM FRAX486 were effective in cells, but not in tissues) may be explained by different content of PAK1, which may be higher in WPMY-1 cells than in prostate tissues.

Our findings with PAK inhibitors in WPMY-1 cells are in line with previous observations. Thus, a critical role of PAKs for actin organization, including polymerization and its organization to filaments, as well as a role for actin-dependent functions such as migration, morphology, proliferation, or contraction was observed in various cell types [35, 44–47, 72]. Deorganization of actin filaments, going along with depolymerization, shortening of filaments, and alterations in morphology can be seen in our phalloidin stainings of WPMY-1 cells. Similar to our observation that cell cycle in prostate stromal cells is PAK-dependent, PAKs were previously found to promote proliferation in airway and vascular smooth muscle cells [22–25]. In the cardiovascular system, PAK1 participates in vascular remodeling, so that an analog role in hyperplastic prostate growth appears now possible [22, 24, 73]. Due to their shared contributions to etiology of voiding symptoms, links between smooth muscle contraction and hyperplastic growth in the prostate have been assumed [2, 5]. Notably, our findings suggest that PAKs may be activated by dihydrostestosterone, which is critical in prostate growth and hyperplasia. This may support the idea, that PAKs are involved in hyperplastic growth besides their role for contraction. Obviously, hyperplastic growth and contraction in BPH are no separate phenomenons, but synchronized and connected with each other by shared molecular mediators, including PAKs.

## Conclusions

Here, we show that control of smooth muscle contraction and stromal growth in the human prostate may be both susceptible to small molecule PAK inhibitors. PAK inhibitors inhibit prostate smooth muscle contraction, and growth of prostate cells, which may both contribute to voiding symptoms. Our findings show that simultaneous targeting of contraction and growth in the prostate by single compounds is principally possible.

## Supporting Information

S1 Fig Complete lanes from Western blot analyses of human prostate tissues (A) and WPMY-1 cells (B) using two different isoform-specific PAK antibodies for each isoform. In **(A)** and **(B)**, upper panels show analyses using polyclonal rabbit or goat antibodies, while monoclonal mouse antibodies were applied in analyses of lower panels. Shown is the total range of membranes being exposed to detection. Numbers left to blots indicate molecular weights (kDa), obtained by a commercially available prestained marker, with the 75 kDa band being stressed and aligned in each line. Arrows indicate bands matching the expected molecular weight of PAK isoforms; if antibodies did not yield bands with expected sizes, arrows indicate the regions where correct bands should be expected.
(TIF)

## References

[1] Gratzke C, Bachmann A, Descazeaud A, Drake MJ, Madersbacher S, Mamoulakis C, et al EAU Guidelines on the Assessment of Non-neurogenic Male Lower Urinary Tract Symptoms including Benign Prostatic Obstruction. Eur Urol. 2015;in press. Epub 2015/01/24. S0302-2838(14)01394-3 [pii] 10.1016/j.eururo.2014.12.038 . 25613154

[2] Hennenberg M, Stief CG, Gratzke C. Prostatic alpha1-adrenoceptors: new concepts of function, regulation, and intracellular signaling. Neurourol Urodyn. 2014;33(7):1074–85. Epub 2013/09/17. 10.1002/nau.22467 . 24038148

[3] Oelke M, Bachmann A, Descazeaud A, Emberton M, Gravas S, Michel MC, et al EAU guidelines on the treatment and follow-up of non-neurogenic male lower urinary tract symptoms including benign prostatic obstruction. Eur Urol. 2013;64(1):118–40. Epub 2013/04/02. S0302-2838(13)00228-5 [pii] 10.1016/j.eururo.2013.03.004 . 23541338

[4] Fullhase C, Chapple C, Cornu JN, De Nunzio C, Gratzke C, Kaplan SA, et al Systematic review of combination drug therapy for non-neurogenic male lower urinary tract symptoms. Eur Urol. 2013;64(2):228–43. Epub 2013/02/05. S0302-2838(13)00030-4 [pii] 10.1016/j.eururo.2013.01.018 . 23375241

[5] Andersson KE, Gratzke C. Pharmacology of alpha1-adrenoceptor antagonists in the lower urinary tract and central nervous system. Nat Clin Pract Urol. 2007;4(7):368–78. Epub 2007/07/07. ncpuro0836 [pii] 10.1038/ncpuro0836 . 17615548

[6] Kunit T, Gratzke C, Schreiber A, Strittmatter F, Waidelich R, Rutz B, et al Inhibition of smooth muscle force generation by focal adhesion kinase inhibitors in the hyperplastic human prostate. Am J Physiol Renal Physiol. 2014;307(7):F823–32. Epub 2014/07/25. ajprenal.00011.2014 [pii] 10.1152/ajprenal.00011.2014 . 25056351

[7] Strittmatter F, Walther S, Gratzke C, Gottinger J, Beckmann C, Roosen A, et al Inhibition of adrenergic human prostate smooth muscle contraction by the inhibitors of c-Jun N-terminal kinase, SP600125 and BI-78D3. Br J Pharmacol. 2012;166(6):1926–35. 10.1111/j.1476-5381.2012.01919.x 22364229 PMC3402815

[8] Wang Y, Kunit T, Ciotkowska A, Rutz B, Schreiber A, Strittmatter F, et al Inhibition of prostate smooth muscle contraction and prostate stromal cell growth by the inhibitors of Rac, NSC23766 and EHT1864. Br J Pharmacol. 2015;in press. Epub 2015/01/30. 10.1111/bph.13099 . 25631101 PMC4439884

[9] Vidal C, Geny B, Melle J, Jandrot-Perrus M, Fontenay-Roupie M. Cdc42/Rac1-dependent activation of the p21-activated kinase (PAK) regulates human platelet lamellipodia spreading: implication of the cortical-actin binding protein cortactin. Blood. 2002;100(13):4462–9. 10.1182/blood.V100.13.4462 . 12453877

[10] Rane CK, Minden A. P21 activated kinases: structure, regulation, and functions. Small GTPases. 2015;5 Epub 2014/03/25. 28003 [pii] 24658305 10.4161/sgtp.28003 PMC4160339

[11] Wen YY, Wang XX, Pei DS, Zheng JN. p21-Activated kinase 5: a pleiotropic kinase. Bioorg Med Chem Lett. 2013;23(24):6636–9. Epub 2013/11/13. S0960-894X(13)01257-2 [pii] 10.1016/j.bmcl.2013.10.051 . 24215894

[12] Chan PM, Manser E. PAKs in human disease. Prog Mol Biol Transl Sci. 2012;106:171–87. Epub 2012/02/22. B978-0-12-396456-4.00011–0 [pii] 10.1016/B978-0-12-396456-4.00011-0 . 22340718

[13] Chu J, Pham NT, Olate N, Kislitsyna K, Day MC, LeTourneau PA, et al Biphasic regulation of myosin light chain phosphorylation by p21-activated kinase modulates intestinal smooth muscle contractility. J Biol Chem. 2013;288(2):1200–13. Epub 2012/11/20. M112.370718 [pii] 10.1074/jbc.M112.370718 23161543 PMC3543003

[14] Foster DB, Shen LH, Kelly J, Thibault P, Van Eyk JE, Mak AS. Phosphorylation of caldesmon by p21-activated kinase. Implications for the Ca(2+) sensitivity of smooth muscle contraction. J Biol Chem. 2000;275(3):1959–65. Epub 2000/01/15. . 10636898 10.1074/jbc.275.3.1959

[15] Gerthoffer WT. Actin cytoskeletal dynamics in smooth muscle contraction. Can J Physiol Pharmacol. 2005;83(10):851–6. Epub 2005/12/08. y05-088 [pii] 10.1139/y05-088 . 16333356

[16] McFawn PK, Shen L, Vincent SG, Mak A, Van Eyk JE, Fisher JT. Calcium-independent contraction and sensitization of airway smooth muscle by p21-activated protein kinase. Am J Physiol Lung Cell Mol Physiol. 2003;284(5):L863–70. Epub 2003/01/07. 10.1152/ajplung.00068.2002 00068.2002 [pii]. . 12513968

[17] Somlyo AP, Somlyo AV. Ca2+ sensitivity of smooth muscle and nonmuscle myosin II: modulated by G proteins, kinases, and myosin phosphatase. Physiol Rev. 2003;83(4):1325–58. Epub 2003/09/25. 10.1152/physrev.00023.2003 83/4/1325 [pii]. . 14506307

[18] Van Eyk JE, Arrell DK, Foster DB, Strauss JD, Heinonen TY, Furmaniak-Kazmierczak E, et al Different molecular mechanisms for Rho family GTPase-dependent, Ca2+-independent contraction of smooth muscle. J Biol Chem. 1998;273(36):23433–9. Epub 1998/08/29. . 9722579 10.1074/jbc.273.36.23433

[19] Wang R, Li QF, Anfinogenova Y, Tang DD. Dissociation of Crk-associated substrate from the vimentin network is regulated by p21-activated kinase on ACh activation of airway smooth muscle. Am J Physiol Lung Cell Mol Physiol. 2007;292(1):L240–8. Epub 2006/09/26. 00199.2006 [pii] 10.1152/ajplung.00199.2006 16997882 PMC1769421

[20] Wirth A, Schroeter M, Kock-Hauser C, Manser E, Chalovich JM, De Lanerolle P, et al Inhibition of contraction and myosin light chain phosphorylation in guinea-pig smooth muscle by p21-activated kinase 1. J Physiol. 2003;549(Pt 2):489–500. Epub 2003/04/15. 10.1113/jphysiol.2002.033167 2002.033167 [pii]. 12692179 PMC2342940

[21] Zhang W, Huang Y, Wu Y, Gunst SJ. A novel role for RhoA GTPase in the regulation of airway smooth muscle contraction. Can J Physiol Pharmacol. 2015;93(2):129–36. Epub 2014/12/23. 10.1139/cjpp-2014-0388 25531582 PMC4399233

[22] Diebold I, Petry A, Djordjevic T, Belaiba RS, Fineman J, Black S, et al Reciprocal regulation of Rac1 and PAK-1 by HIF-1alpha: a positive-feedback loop promoting pulmonary vascular remodeling. Antioxid Redox Signal. 2010;13(4):399–412. Epub 2009/12/17. 10.1089/ars.2009.3013 . 20001745

[23] Hirst SJ, Martin JG, Bonacci JV, Chan V, Fixman ED, Hamid QA, et al Proliferative aspects of airway smooth muscle. J Allergy Clin Immunol. 2004;114(2 Suppl):S2–17. Epub 2004/08/17. 10.1016/j.jaci.2004.04.039 S0091674904014125 [pii]. . 15309015

[24] Kundumani-Sridharan V, Singh NK, Kumar S, Gadepalli R, Rao GN. Nuclear factor of activated T cells c1 mediates p21-activated kinase 1 activation in the modulation of chemokine-induced human aortic smooth muscle cell F-actin stress fiber formation, migration, and proliferation and injury-induced vascular wall remodeling. J Biol Chem. 2013;288(30):22150–62. Epub 2013/06/06. M113.454082 [pii] 10.1074/jbc.M113.454082 23737530 PMC3724667

[25] Toumaniantz G, Ferland-McCollough D, Cario-Toumaniantz C, Pacaud P, Loirand G. The Rho protein exchange factor Vav3 regulates vascular smooth muscle cell proliferation and migration. Cardiovasc Res. 2010;86(1):131–40. Epub 2009/12/09. cvp387 [pii] 10.1093/cvr/cvp387 . 19969623

[26] Al-Azayzih A, Gao F, Somanath PR. P21 activated kinase-1 mediates transforming growth factor beta1-induced prostate cancer cell epithelial to mesenchymal transition. Biochim Biophys Acta. 2015;1853(5):1229–39. 10.1016/j.bbamcr.2015.02.023 25746720 PMC4380670

[27] Goc A, Al-Azayzih A, Abdalla M, Al-Husein B, Kavuri S, Lee J, et al P21 activated kinase-1 (Pak1) promotes prostate tumor growth and microinvasion via inhibition of transforming growth factor beta expression and enhanced matrix metalloproteinase 9 secretion. J Biol Chem. 2013;288(5):3025–35. 10.1074/jbc.M112.424770 23258534 PMC3561527

[28] Kaur R, Yuan X, Lu ML, Balk SP. Increased PAK6 expression in prostate cancer and identification of PAK6 associated proteins. Prostate. 2008;68(14):1510–6. 10.1002/pros.20787 . 18642328

[29] Park MH, Lee HS, Lee CS, You ST, Kim DJ, Park BH, et al p21-Activated kinase 4 promotes prostate cancer progression through CREB. Oncogene. 2013;32(19):2475–82. 10.1038/onc.2012.255 . 22710715

[30] Shin YJ, Kim EH, Roy A, Kim JH. Evidence for a novel mechanism of the PAK1 interaction with the Rho-GTPases Cdc42 and Rac. PLoS One. 2013;8(8):e71495 10.1371/journal.pone.0071495 23936510 PMC3731272

[31] Pradidarcheep W, Wallner C, Dabhoiwala NF, Lamers WH. Anatomy and histology of the lower urinary tract. Handb Exp Pharmacol. 2011;(202):117–48. Epub 2011/02/04. 10.1007/978-3-642-16499-6_7 . 21290225

[32] Shaikhibrahim Z, Lindstrot A, Ellinger J, Rogenhofer S, Buettner R, Perner S, et al The peripheral zone of the prostate is more prone to tumor development than the transitional zone: is the ETS family the key? Mol Med Rep. 2012;5(2):313–6. Epub 2011/11/01. 10.3892/mmr.2011.647 . 22038307

[33] Alcaraz A, Hammerer P, Tubaro A, Schroder FH, Castro R. Is there evidence of a relationship between benign prostatic hyperplasia and prostate cancer? Findings of a literature review. Eur Urol. 2009;55(4):864–73. Epub 2008/11/26. S0302-2838(08)01315-8 [pii] 10.1016/j.eururo.2008.11.011 . 19027219

[34] Orsted DD, Bojesen SE. The link between benign prostatic hyperplasia and prostate cancer. Nat Rev Urol. 2013;10(1):49–54. Epub 2012/11/21. nrurol.2012.192 [pii] 10.1038/nrurol.2012.192 . 23165396

[35] Kim YB, Shin YJ, Roy A, Kim JH. The Role of the Pleckstrin Homology Domain-containing Protein CKIP-1 in Activation of p21-activated Kinase 1 (PAK1). J Biol Chem. 2015;290(34):21076–85. 10.1074/jbc.M115.675124 26160174 PMC4543665

[36] Deacon SW, Beeser A, Fukui JA, Rennefahrt UE, Myers C, Chernoff J, et al An isoform-selective, small-molecule inhibitor targets the autoregulatory mechanism of p21-activated kinase. Chem Biol. 2008;15(4):322–31. Epub 2008/04/19. S1074-5521(08)00116-6 [pii] 10.1016/j.chembiol.2008.03.005 18420139 PMC4353635

[37] Yu JS, Chen WJ, Ni MH, Chan WH, Yang SD. Identification of the regulatory autophosphorylation site of autophosphorylation-dependent protein kinase (auto-kinase). Evidence that auto-kinase belongs to a member of the p21-activated kinase family. Biochem J. 1998;334 (Pt 1):121–31. 9693111 10.1042/bj3340121 PMC1219670

[38] Levitt JM, Slawin KM. Prostate-specific antigen and prostate-specific antigen derivatives as predictors of benign prostatic hyperplasia progression. Curr Urol Rep. 2007;8(4):269–74. Epub 2008/06/04. . 18519010 10.1007/s11934-007-0072-y

[39] Coleman N, Kissil J. Recent advances in the development of p21-activated kinase inhibitors. Cell Logist. 2012;2(2):132–5. Epub 2012/11/20. 10.4161/cl.21667 2012CELLULARLOG0022R [pii]. 23162744 PMC3490963

[40] Dolan BM, Duron SG, Campbell DA, Vollrath B, Shankaranarayana Rao BS, Ko HY, et al Rescue of fragile X syndrome phenotypes in Fmr1 KO mice by the small-molecule PAK inhibitor FRAX486. Proc Natl Acad Sci U S A. 2013;110(14):5671–6. Epub 2013/03/20. 1219383110 [pii] 10.1073/pnas.1219383110 23509247 PMC3619302

[41] Viaud J, Peterson JR. An allosteric kinase inhibitor binds the p21-activated kinase autoregulatory domain covalently. Mol Cancer Ther. 2009;8(9):2559–65. Epub 2009/09/03. 1535-7163.MCT-09-0102 [pii] 10.1158/1535-7163.MCT-09-0102 19723886 PMC2782767

[42] Davis B, Goepel M, Bein S, Chess-Williams R, Chapple CR, Michel MC. Lack of neuropeptide Y receptor detection in human bladder and prostate. BJU Int. 2000;85(7):918–24. . 10792177 10.1046/j.1464-410x.2000.00573.x

[43] Guh JH, Ko FN, Chueh SC, Lai MK, Teng CM. Ouabain-induced increases in resting tone of human hyperplastic prostate following repeated noradrenaline and electrical field stimulation. Br J Pharmacol. 1996;117(8):1716–20. 8732281 10.1111/j.1476-5381.1996.tb15344.x PMC1909572

[44] Baek SH, Cho HW, Kwon YC, Lee JH, Kim MJ, Lee H, et al Requirement for Pak3 in Rac1-induced organization of actin and myosin during Drosophila larval wound healing. FEBS Lett. 2012;586(6):772–7. 10.1016/j.febslet.2012.01.061 . 22449966

[45] Garcia M, Ray S, Brown I, Irom J, Brazill D. PakD, a putative p21-activated protein kinase in Dictyostelium discoideum, regulates actin. Eukaryot Cell. 2014;13(1):119–26. 10.1128/EC.00216-13 24243792 PMC3910960

[46] Holderness Parker N, Donninger H, Birrer MJ, Leaner VD. p21-activated kinase 3 (PAK3) is an AP-1 regulated gene contributing to actin organisation and migration of transformed fibroblasts. PLoS One. 2013;8(6):e66892 10.1371/journal.pone.0066892 23818969 PMC3688571

[47] Van den Broeke C, Radu M, Deruelle M, Nauwynck H, Hofmann C, Jaffer ZM, et al Alphaherpesvirus US3-mediated reorganization of the actin cytoskeleton is mediated by group A p21-activated kinases. Proc Natl Acad Sci U S A. 2009;106(21):8707–12. 10.1073/pnas.0900436106 19435845 PMC2688975

[48] Khare V, Dammann K, Asboth M, Krnjic A, Jambrich M, Gasche C. Overexpression of PAK1 promotes cell survival in inflammatory bowel diseases and colitis-associated cancer. Inflamm Bowel Dis. 2015;21(2):287–96. 10.1097/MIB.0000000000000281 25569743 PMC4345971

[49] Ong CC, Gierke S, Pitt C, Sagolla M, Cheng CK, Zhou W, et al Small molecule inhibition of group I p21-activated kinases in breast cancer induces apoptosis and potentiates the activity of microtubule stabilizing agents. Breast Cancer Res. 2015;17:59 10.1186/s13058-015-0564-5 25902869 PMC4445529

[50] Zeng Y, Broxmeyer HE, Staser K, Chitteti BR, Park SJ, Hahn S, et al Pak2 regulates hematopoietic progenitor cell proliferation, survival, and differentiation. Stem Cells. 2015;33(5):1630–41. 10.1002/stem.1951 25586960 PMC4409559

[51] Chapple CR, Montorsi F, Tammela TL, Wirth M, Koldewijn E, Fernandez Fernandez E. Silodosin therapy for lower urinary tract symptoms in men with suspected benign prostatic hyperplasia: results of an international, randomized, double-blind, placebo- and active-controlled clinical trial performed in Europe. Eur Urol. 2011;59(3):342–52. Epub 2010/11/27. S0302-2838(10)01058-4 [pii] 10.1016/j.eururo.2010.10.046 . 21109344

[52] Kortmann BB, Floratos DL, Kiemeney LA, Wijkstra H, de la Rosette JJ. Urodynamic effects of alpha-adrenoceptor blockers: a review of clinical trials. Urology. 2003;62(1):1–9. Epub 2003/07/03. S0090429502021131 [pii]. . 12837408 10.1016/s0090-4295(02)02113-1

[53] Lee HN, Lee KS, Kim JC, Chung BH, Kim CS, Lee JG, et al Rate and associated factors of solifenacin add-on after tamsulosin monotherapy in men with voiding and storage lower urinary tract symptoms. Int J Clin Pract. 2015;69(4):444–53. Epub 2014/11/05. 10.1111/ijcp.12581 . 25363606

[54] Madersbacher S, Marszalek M, Lackner J, Berger P, Schatzl G. The long-term outcome of medical therapy for BPH. Eur Urol. 2007;51(6):1522–33. Epub 2007/04/10. S0302-2838(07)00434-4 [pii] 10.1016/j.eururo.2007.03.034 . 17416456

[55] Matsukawa Y, Gotoh M, Komatsu T, Funahashi Y, Sassa N, Hattori R. Efficacy of silodosin for relieving benign prostatic obstruction: prospective pressure flow study. J Urol. 2013;189(1 Suppl):S117–21. Epub 2012/12/19. S0022-5347(12)05496-1 [pii] 10.1016/j.juro.2012.11.031 . 23234615

[56] Cindolo L, Pirozzi L, Fanizza C, Romero M, Tubaro A, Autorino R, et al Drug Adherence and Clinical Outcomes for Patients Under Pharmacological Therapy for Lower Urinary Tract Symptoms Related to Benign Prostatic Hyperplasia: Population-based Cohort Study. Eur Urol. 2013 Epub 2014/12/04. S0302-2838(14)01180-4 [pii] 10.1016/j.eururo.2014.11.006 . 25465970

[57] Hayashi-Takagi A, Araki Y, Nakamura M, Vollrath B, Duron SG, Yan Z, et al PAKs inhibitors ameliorate schizophrenia-associated dendritic spine deterioration in vitro and in vivo during late adolescence. Proc Natl Acad Sci U S A. 2014;111(17):6461–6. Epub 2014/04/08. 1321109111 [pii] 10.1073/pnas.1321109111 24706880 PMC4035976

[58] Tilki D, Mandel P, Seeliger F, Kretschmer A, Karl A, Ergun S, et al Salvage lymph node dissection for nodal recurrence of prostate cancer after radical prostatectomy. J Urol. 2015;193(2):484–90. 10.1016/j.juro.2014.08.096 . 25180792

[59] Khoder WY, Waidelich R, Buchner A, Becker AJ, Stief CG. Prospective comparison of one year follow-up outcomes for the open complete intrafascial retropubic versus interfascial nerve-sparing radical prostatectomy. Springerplus. 2014;3:335 10.1186/2193-1801-3-335 25032093 PMC4094758

[60] Khoder WY, Schlenker B, Waidelich R, Buchner A, Kellhammer N, Stief CG, et al Open complete intrafascial nerve-sparing retropubic radical prostatectomy: technique and initial experience. Urology. 2012;79(3):717–21. 10.1016/j.urology.2011.11.045 . 22386427

[61] Wells CM, Jones GE. The emerging importance of group II PAKs. Biochem J. 2010;425(3):465–73. Epub 2010/01/15. BJ20091173 [pii] 10.1042/BJ20091173 . 20070256

[62] Chahdi A, Miller B, Sorokin A. Endothelin 1 induces beta 1Pix translocation and Cdc42 activation via protein kinase A-dependent pathway. J Biol Chem. 2005;280(1):578–84. 10.1074/jbc.M411130200 . 15513924

[63] Hennenberg M, Miljak M, Herrmann D, Strittmatter F, Walther S, Rutz B, et al The receptor antagonist picotamide inhibits adrenergic and thromboxane-induced contraction of hyperplastic human prostate smooth muscle. Am J Physiol Renal Physiol. 2013;305(10):F1383–90. Epub 2013/09/21. ajprenal.00380.2013 [pii] 10.1152/ajprenal.00380.2013 . 24049147

[64] Raymond K, Cagnet S, Kreft M, Janssen H, Sonnenberg A, Glukhova MA. Control of mammary myoepithelial cell contractile function by alpha3beta1 integrin signalling. EMBO J. 2011;30(10):1896–906. 10.1038/emboj.2011.113 21487391 PMC3098485

[65] Li QF, Tang DD. Role of p47(phox) in regulating Cdc42GAP, vimentin, and contraction in smooth muscle cells. Am J Physiol Cell Physiol. 2009;297(6):C1424–33. 10.1152/ajpcell.00324.2009 19812368 PMC2793062

[66] Li QF, Spinelli AM, Tang DD. Cdc42GAP, reactive oxygen species, and the vimentin network. Am J Physiol Cell Physiol. 2009;297(2):C299–309. 10.1152/ajpcell.00037.2009 19494238 PMC2724092

[67] Ohtakara K, Inada H, Goto H, Taki W, Manser E, Lim L, et al p21-activated kinase PAK phosphorylates desmin at sites different from those for Rho-associated kinase. Biochem Biophys Res Commun. 2000;272(3):712–6. 10.1006/bbrc.2000.2854 . 10860820

[68] Goeckeler ZM, Masaracchia RA, Zeng Q, Chew TL, Gallagher P, Wysolmerski RB. Phosphorylation of myosin light chain kinase by p21-activated kinase PAK2. J Biol Chem. 2000;275(24):18366–74. 10.1074/jbc.M001339200 . 10748018

[69] Delorme-Walker VD, Peterson JR, Chernoff J, Waterman CM, Danuser G, DerMardirossian C, et al Pak1 regulates focal adhesion strength, myosin IIA distribution, and actin dynamics to optimize cell migration. J Cell Biol. 2011;193(7):1289–303. 10.1083/jcb.201010059 21708980 PMC3216326

[70] Zhang H, Landmann F, Zahreddine H, Rodriguez D, Koch M, Labouesse M. A tension-induced mechanotransduction pathway promotes epithelial morphogenesis. Nature. 2011;471(7336):99–103. 10.1038/nature09765 . 21368832

[71] Fediuk J, Sikarwar AS, Nolette N, Dakshinamurti S. Thromboxane-induced actin polymerization in hypoxic neonatal pulmonary arterial myocytes involves Cdc42 signaling. Am J Physiol Lung Cell Mol Physiol. 2014;307(11):L877–87. 10.1152/ajplung.00036.2014 . 25281640

[72] Even-Faitelson L, Rosenberg M, Ravid S. PAK1 regulates myosin II-B phosphorylation, filament assembly, localization and cell chemotaxis. Cell Signal. 2005;17(9):1137–48. 10.1016/j.cellsig.2004.12.015 . 15993754

[73] Hinoki A, Kimura K, Higuchi S, Eguchi K, Takaguri A, Ishimaru K, et al p21-activated kinase 1 participates in vascular remodeling in vitro and in vivo. Hypertension. 2010;55(1):161–5. Epub 2009/11/11. HYPERTENSIONAHA.109.143057 [pii] 10.1161/HYPERTENSIONAHA.109.143057 19901155 PMC2810611

