# Supplementary figures and images for: P21-Activated Kinase Inhibitors FRAX486 and IPA3: Inhibition of Prostate Stromal Cell Growth and Effects on Smooth Muscle Contraction in the Human Prostate
Source: PLoS One. 2016 Apr 12;11(4):e0153312. doi: 10.1371/journal.pone.0153312 (PMC4829229; doi:10.1371/journal.pone.0153312)

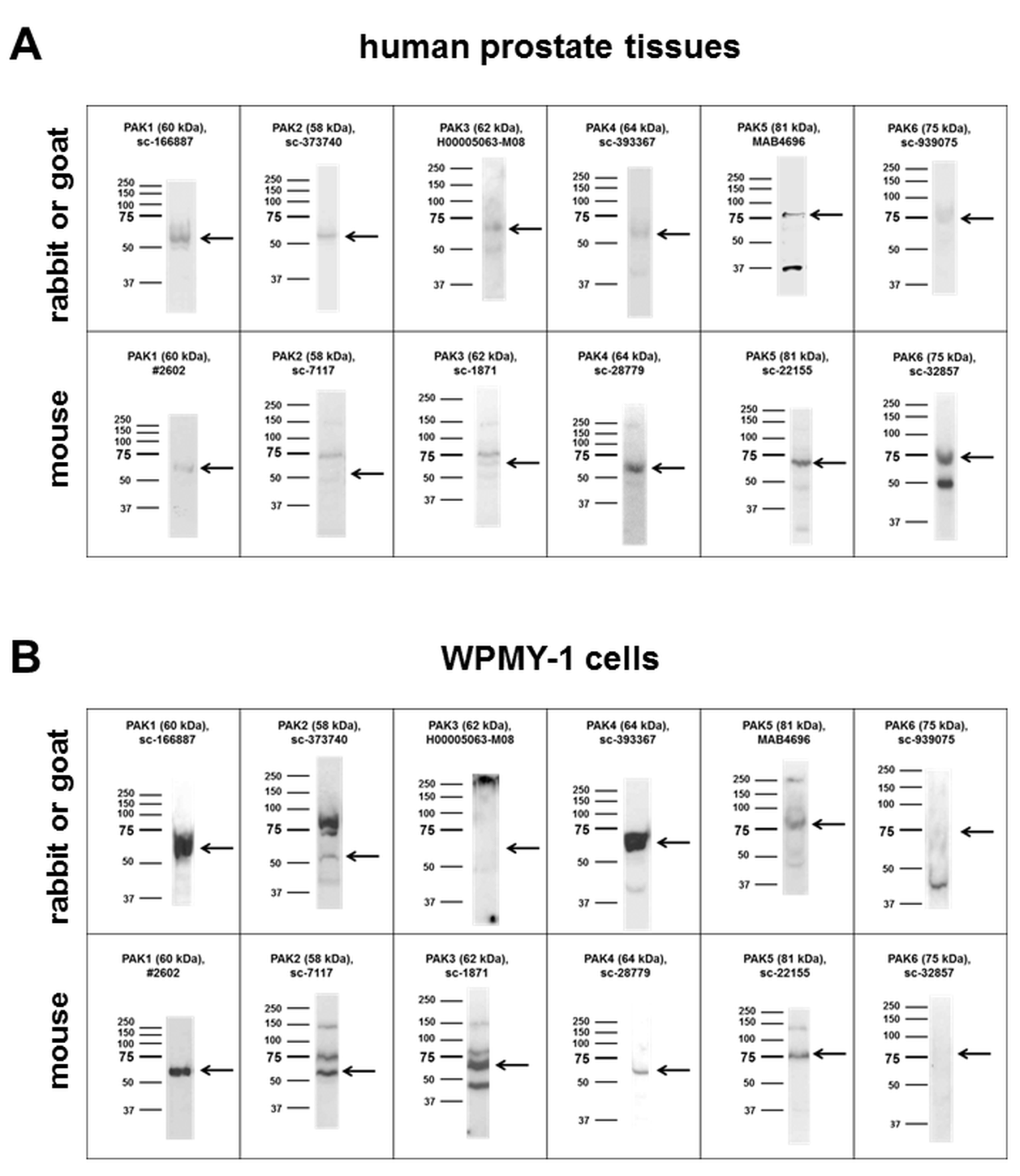

Supplement: S1 Fig — In (A) and (B), upper panels show analyses using polyclonal rabbit or goat antibodies, while monoclonal mouse antibodies were applied in analyses of lower panels. Shown is the total range of membranes being exposed to detection. Numbers left to blots indicate molecular weights (kDa), obtained by a commercially available prestained marker, with the 75 kDa band being stressed and aligned in each line. Arrows indicate bands matching the expected molecular weight of PAK isoforms; if antibodies did not yield bands with expected sizes, arrows indicate the regions where correct bands should be expected. (TIF) [file pone.0153312.s001.tif]
